# Cu Transport by the Extended Family of CcoA-like Transporters (CalT) in Proteobacteria

**DOI:** 10.1038/s41598-018-37988-4

**Published:** 2019-02-04

**Authors:** Yang Zhang, Crysten E. Blaby-Haas, Stefan Steimle, Andreia F. Verissimo, Victor A. Garcia-Angulo, Hans-Georg Koch, Fevzi Daldal, Bahia Khalfaoui-Hassani

**Affiliations:** 1 Department of Biology, University of Pennsylvania, Philadelphia, PA 19104 USA; 2 Biology Department, Brookhaven National Laboratory, Upton, NY 11973 USA; 3 Microbiology and Mycology Department, Insituto de Ciencias Biomédicas, University of Chile, Santiago, Chile; 4 Institut für Biochemie und Molekularbiologie, ZBMZ, Faculty of Medicine, Stefan-Meier-Strasse 17, Albert-Ludwigs-Universität Freiburg, 79104 Freiburg, Germany; 5 Present Address: IPREM, UMR CNRS 5254, and Université de Pau et des Pays de l’Adour, BP1155 Pau, France; 6 Present Address: School of Life Science, Xiamen University, Xiamen, 361102 China; 7 Present Address: bioMT-Institute for Biomolecular Targeting, Geisel School of Medicine at Dartmouth, Hanover, NH 03755 USA

## Abstract

Comparative genomic studies of the bacterial MFS-type copper importer CcoA, required for *cbb*_3_-type cytochrome *c* oxidase (*cbb*_3_-Cox) biogenesis, revealed a widespread **C**co**A**-**l**ike **t**ransporters (CalT) family, containing the conserved CcoA Cu-binding **M**xxx**M** and **H**xxx**M** motifs. Surprisingly, this family also included the RfnT-like proteins, earlier suggested to transport riboflavin. However, presence of the Cu-binding motifs in these proteins raised the possibility that they might be Cu transporters. To test this hypothesis, the genomic context of the corresponding genes was examined, and three of such genes from *Ochrobactrum anthropi*, *Rhodopseudomonas palustris* and *Agrobacterium tumefaciens* were expressed in *Escherichia coli* (Δ*ribB*) and *Rhodobacter capsulatus* (Δ*ccoA*) mutants. Copper and riboflavin uptake abilities of these strains were compared with those expressing *R. capsulatus* CcoA and *Rhizobium leguminosarum* RibN as *bona fide* copper and riboflavin importers, respectively. Overall data demonstrated that the “RfnT-like” CalT proteins are unable to efficiently transport riboflavin, but they import copper like CcoA. Nevertheless, even though expressed and membrane-localized in a *R. capsulatus* mutant lacking CcoA, these transporters were unable to accumulate Cu or complement for *cbb*_3_-Cox defect. This lack of functional exchangeability between the different subfamilies of CalT homologs suggests that MFS-type bacterial copper importers might be species-specific.

## Introduction

The major facilitator superfamily (MFS) transporters are ubiquitous to all branches of life, and play central roles in various biological processes. This superfamily is one of the largest group of secondary active transporters that selectively transport a remarkable variety of substrates across cell membranes (http://www.tcdb.org)^1^. Their substrates include mono- and oligo-saccharides, peptides, drugs, siderophores, metal ions as well as inorganic anions and cations^2,3^. Based on the nature of the substrates and modes of transport (uniporters, symporters and antiporters), the MFS superfamily members are divided into several families, according to the Transporter Classification Database (TCDB, http://www.tcdb.org). The primary amino acid sequences of these transporters are not very similar, yet they all share universally conserved (GxxxDxxxxxRxGRR and RxxxG) motifs^4^. Although the number of transmembrane (TM) helices varies among members of the superfamily^5^, most MFS transporters are composed of 12 TM helices^6–8^ resulting from two successive duplications of a three-TM-core. It has been hypothesized that these proteins function via an “alternating access” mechanism^9,10^, which postulates that the transporter switches between two conformations, allowing the substrate to access the binding site embedded in one side of the membrane. Substrate binding to the transporter in an outward-open conformation induces structural changes, switching it to an inward-open form, which allows release of the substrate to the opposite side of the membrane^10,11^.

The first bacterial copper (Cu) importer of MFS-type, CcoA (RCC02192) was discovered in the facultative photosynthetic model organism *Rhodobacter capsulatus*^12,13^ and since then has become the prototype of the newly defined Copper Uptake Porter Family (TCDB: 2.A1.81.1^1^). Earlier genetic and biochemical studies unequivocally established that CcoA imports Cu destined to the catalytic site (Cu_B_ center) of the *cbb*_3_-type cytochrome *c* oxidases (*cbb*_3_-Cox), where O_2_ is reduced to H_2_O during aerobic respiration^14–16^. Subsequent studies identified putative Cu-binding sites of CcoA, involving the **M**xxx**M** and **H**xxx**M** motifs located at its TM7 and TM8^16^. Our previous comparative genomics of CcoA homologs revealed the existence of an extensive family of CcoA-like MFS-type transporters, ubiquitous among bacteria and also present in a few microbial eukaryotes^17^. Moreover, the corresponding genes often co-occur with genes encoding *aa*_3_- and *cbb*_3_-Cox in alpha-proteobacterial genomes, but they do not appear to provide Cu to all cupro-enzymes. In *Rhodobacter sphaeroides*, CcoA is required only for *cbb*_3_- but not for *aa*_3_-Cox biogenesis even though both enzymes have quasi-identical Cu_B_ centers^17^. In the present study, we refer to this large family of proteins as the **C**co**A**-**l**ike **t**ransporters or CalT.

Unexpectedly, the protein similarity network analysis of the CalT family identified a subgroup of organisms that contain protein sequences^17^ previously annotated as riboflavin transporters (RfnT)^18^. The amino acid sequences from this “RfnT-like” CalT subgroup are highly similar to *R. capsulatus* and *R. sphaeroides* CcoA and also contain the putative Cu binding motifs (**M**xxx**M** and **H**xxx**M)**^16^. In some species, like *Mesorhizobium loti, Sinorhizobium meliloti* and *Agrobacterium tumafeciens*, the *rfnT* gene is the last open reading frame (ORF) of a hypothetical riboflavin biosynthesis pathway (RBP) operon (*nrdR-ribDEH-nusB-rfnT*)^18^. Based on the positional clustering of *rfnT* with the RBP genes and its homology to membrane transporters, these proteins were suggested to be riboflavin-related transporters. Later, *Ochrobactrum anthropi rfnT* was introduced into an *E. coli* riboflavin auxotrophic mutant (Δ*ribB*) conferring the ability to grow in the presence of low amounts (2.5 μM) of riboflavin, which led to the suggestion that *O. anthropi* RfnT was a riboflavin transporter^19,20^. Surprisingly, the presence of a riboflavin-responsive riboswitch upstream of *nrdR-ribDEH-nusB-rfnT* has not been found so far in analyzed genomes^19–21^. Also, two genes of this cluster, *nrdR* and *nusB*, which function as a transcriptional repressor of ribonucleotide reductases and a factor of the bacterial anti-terminator complex, respectively, are not related to riboflavin biosynthesis, further bringing into question the role of RfnT proteins in riboflavin transport.

In this work, we performed a phylogenomic analysis to determine whether RfnT defines a functionally different subgroup of the CalT family, with distinct substrate specificity. To this end, we examined the genomic context of CalT genes, and expressed three RfnT-like CalT homologs from *O. anthropi* (*calT*-O: OANT_22955, formerly called RfnT^19^), *A. tumefaciens* (*calT*-A: ATU_1173) and *Rhodopseudomonas palustris* (*calT*-R: TX73-RS24555) in *E. coli* Δ*ribB* and *R. capsulatus* Δ*ccoA* mutants, and investigated their ability to transport Cu or riboflavin. The overall data supported the conclusion that the RfnT-like CalT subfamily members imported Cu like CcoA, and not riboflavin unlike RibN. The findings showed that the conserved **M**xxx**M** and **H**xxx**M** motifs are strong predictors of Cu-transport activity, and suggested that members of the CalT family may exhibit species-specificity for Cu uptake, illustrating the ability of different organisms to use dedicated Cu uptake and delivery pathways to channel Cu to target proteins.

## Results

### Amino acid sequence similarity analyses of CcoA-like transporters (CalT)

**Figure 1:**
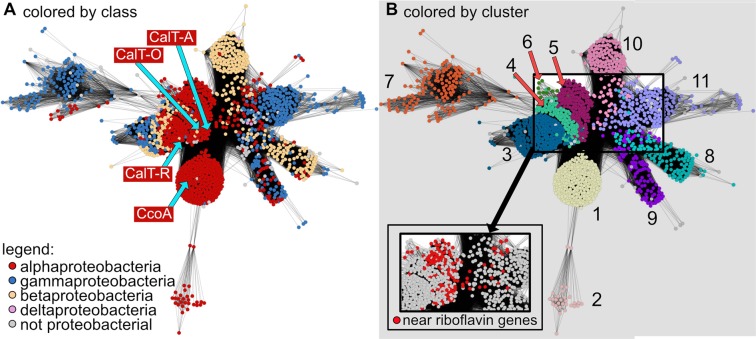
CcoA-like transporter (CalT) Protein Similarity Network. A protein similarity network of MFS members that are homologous to CcoA is shown. Each node (circle) represents one or more protein sequences, and each edge (solid line) represents similarity between two proteins (threshold set at an alignment score of 75). (**A**) Nodes are colored by taxonomic classes as indicated. The locations of nodes representing proteins examined in this study are indicated with blue arrows. (**B**) Nodes are colored by clusters as found in, and numbered according to Fig. 2A. Nodes that represent proteins encoded by genes found near a riboflavin biosynthetic pathway (RBP) cluster are colored red in the pop-out.

**Figure 2:**
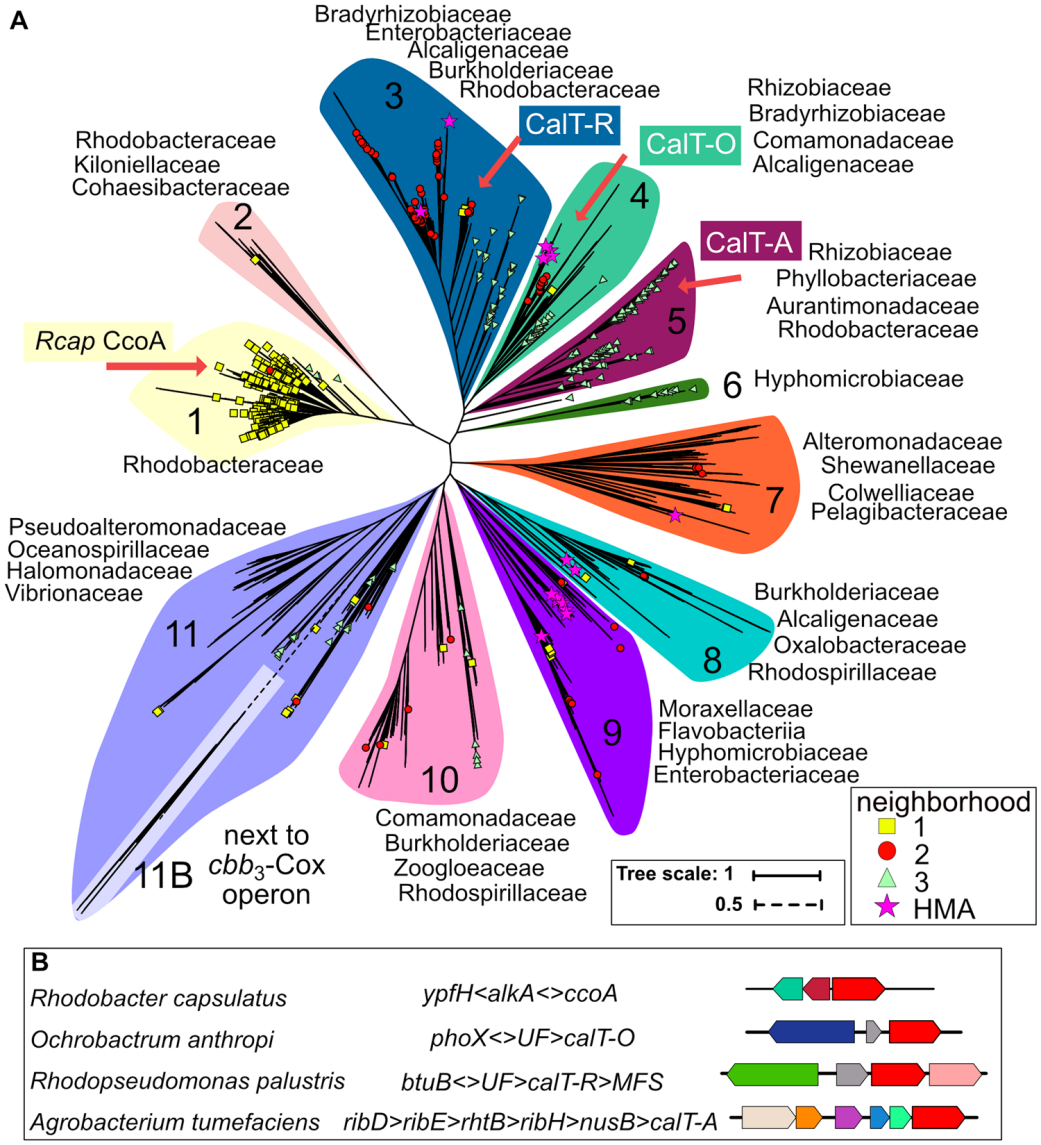
Phylogenetic distribution of CalT in Proteobacteria. (**A**) A phylogenetic tree of proteins from the sequence similarity network, plus 12 *Rhizobiales* sequences that are encoded next to the *cbb*_3_-Cox biogenesis gene cluster, is shown. Background shading corresponds to separate clusters (1 to 11), and leaves corresponding to the proteins experimentally examined in this study are indicated by a red arrow. Whether a CalT is encoded by a gene found in one of the three main genomic neighborhoods is indicated with either a yellow square (N1), a red circle (N2) or a green triangle (N3) according to the legend. A star (Heavy Metal Associated, HMA) indicates that the corresponding gene is found near a putative Cu homeostasis gene. A full list of all proteins analyzed and the related information can be found in SI Table S1. (**B**) Genomic neighborhoods of the RfnT-like CalT proteins examined in this study are compared to *R. capsulatus* CcoA. All gene abbreviations are listed in SI Table S4.

**Figure 3:**
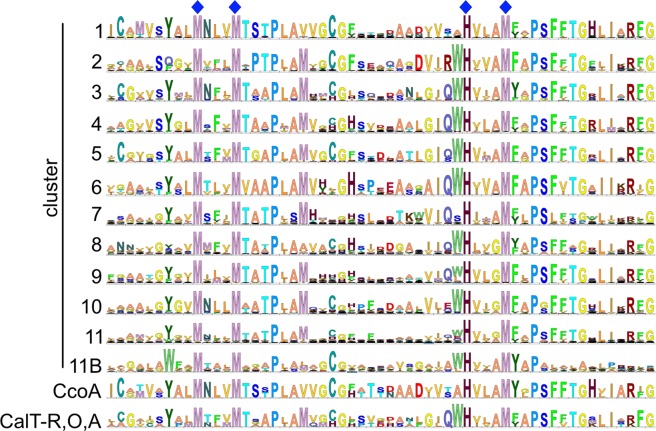
Sequence logos for each CalT cluster. Sequence logo “11B” was constructed using only the MFS proteins that are encoded by the *calT* genes found next to the *cbb*_3_-Cox biogenesis genes cluster. The sequence logo “CcoA” was constructed using CcoA from *R. capsulatus* and *R. sphaeroides*, and the sequence logo “CalT-R, O, A” was built using CalT-O, CalT-R and CalT-A. Dark blue diamond symbols indicate the putative Cu-binding motifs **M**xxx**M** and **H**xxx**M** in TM7 and TM8 of CcoA.

CcoA homologs, referred here as CalT, are found throughout the bacterial kingdom and also encoded in the genomes of some microbial eukaryotes^17^. The vast majority of proteins from each taxonomically distinct subfamily of CalT contain the motifs **M**xxx**M** in TM7 and **H**xxx**M** in TM8, which are required for Cu uptake and *cbb*_3_-Cox biogenesis in *R. capsulatus*^16^. These findings suggest that Cu import might be a ubiquitous function for this family of MFS transporters. As a first step in addressing this hypothesis, we performed a phylogenetic and genomic context analysis on the CalT subfamily members that are mainly from other *Proteobacteria* and exhibit highest similarity to CcoA from *R. capsulatus* and CalT-O (formerly RfnT) from *O. anthropi*, Based on the protein similarity network (Fig. 1) and the phylogenetic tree (Fig. 2A), 11 distinct clusters (numbered 1 to 11, Figs 1B and 2A) were identified, and the amino acid contexts of their conserved **M**xxx**M** and **H**xxx**M** motifs are shown in Fig. 3. The three largest subunits of *cbb*_3_-Cox (CcoN, CcoO and CcoP) were found encoded in most, but not all, of these proteobacterial genomes (SI Fig. 1), suggesting that not all CalT are involved in supplying Cu for *cbb*_3_-Cox biogenesis. The CcoA from *R. capsulatus*^13^ and *R. sphaeroides*^17^ were found in cluster 1, which is shared with orthologous proteins from the *Rhodobacteraceae* family, whereas CalT-O was found in cluster 4 (Fig. 2A). Due to sequence divergence, the *Rhizobiales* CalT proteins which are truncated at the C-terminus and whose corresponding genes are located next to the *cbb*_3_-Cox biogenesis (*ccoNOQP-ccoGHIS*) cluster^17^, were not connected to the network. However, when these sequences were included in the phylogenetic analyses, they were found most closely related to proteins within cluster 11 (Fig. 2A, cluster 11B), instead of cluster 1, which contains members experimentally shown to be required for *cbb*_3_-Cox biogenesis.

### Genomic context of CalT

Next, a neighborhood analysis was performed to identify proteins other than *cbb*_3_-Cox that might be functionally linked to CalT. Functionally coupled genes tend to cluster physically in bacterial genomes, and both frequency and conservation of gene clustering across evolutionarily distant genomes can be used to detect functional coupling^22^. We used a window of three genes upstream and downstream of each *calT* gene encoding a CalT protein from the similarity network to analyze the extent to which the neighboring genes are conserved at the genus, family, and order levels of taxonomy. At the genus level, we identified 605 protein family (Pfam) domains or domain fusions (referred to as neighbors) that were seen in at least two different genera. We ranked these domains by number of genera, excluded putative transcription factors and transporters, and further analyzed the top 17 neighbors (each found in 30 or more genera) (Methods). These neighbors could be arranged into three main neighborhoods (SI Table S1). The first neighborhood N1 (yellow squares in Fig. 2A), which contains CcoA from *Rhodobacter* species, was composed of one or more of nine genes including the putative DNA repair (*alkA*, PF00730) and esterase (*ypfH*, PF02230) proteins (Fig. 2B). The second neighborhood N2 (red circles in Fig. 2A) contained the genes encoding a FabG-like reductase (PF13561) and/or a putative Zn-dependent dehydrogenase (PF00107-PF08240). The third neighborhood N3 (green triangles in Fig. 2A) contained genes encoding a BamE-like outer membrane protein assembly factor (PF04355), a putative ubiquinol-cytochrome *c* oxidoreductase (cytochrome *bc*_1_ complex) chaperone (PF03981), a putative thiamine-monophosphate kinase (PF00586-PF02769) and/or a putative 6,7-dimethyl-8-ribityllumazine synthase (*ribH* involved in riboflavin biosynthesis, PF00885). These main neighborhoods (yellow squares, red circles and green triangles) are indicated in Fig. 2A, and all neighboring genes are listed in SI Table S1 (Tree and Neighborhood sheets). Of the neighborhoods, only N2 and a putative methyl transferase from N1 were enriched at the family and order levels. The RBP protein RibH, BamE-like outer membrane protein assembly factor, and ubiquinol-cytochrome *c* oxidoreductase chaperone from N3 were enriched at the family, but not at the order level (SI Table S1).

### Positional clustering of RfnT-like CalT proteins with RBP genes

The neighborhood N3 captured the positional clustering that originally led to the identification of RfnT in *M. loti, S. meliloti* and *A. tumefaciens*, and prediction that these might be riboflavin transporters^18^. Many more bacterial genomes have been sequenced since that original analysis, and our data show that positional clustering between RBP genes and those encoding RfnT-like CalT proteins is conserved only in *Rhizobiales*, in a small subset of *Rhodobacterales*, and in *Rhodospirillales* (SI Figs S1A and S2A). Current data indicate that the proximity of these *rfnT*-like *calT* to *ribH* is mainly observed in clusters 5 and 6, and near the base of the clusters 3, 4, 10 and 11. The core unit, seen in cluster 5, is composed of the RBP gene *ribH*, followed by *nusB* encoding a subunit of the global transcriptional antitermination complex, and finally by *calT*. In addition, the *thiL* gene encoding thiamine-monophosphate kinase (vitamin B1 biosynthesis) and some other presumably functionally unrelated genes separating *rfnT* from *ribH-nusB* (clusters 3, 4, 10, and 11) were frequently seen (SI Fig. S2B). In most cases, RBP genes other than *ribH* are also conserved upstream of the core *ribH*-*nusB*-*rfnT* unit (SI Fig. S2B). Thus, the genomic proximity of the genes encoding the RfnT-like CalT to RibH-related proteins is not general, but is only seen in a subset of the clusters and in relatively closely related bacteria. Similarly, the previously identified group of *Rhizobiales* CalT is the only example where *calT* was located next to the *cbb*_3_-Cox biogenesis genes (*ccoNOQP-ccoGHIS*)^17^.

### Cu-related proteins found in neighborhoods containing CalT

**Figure 4:**
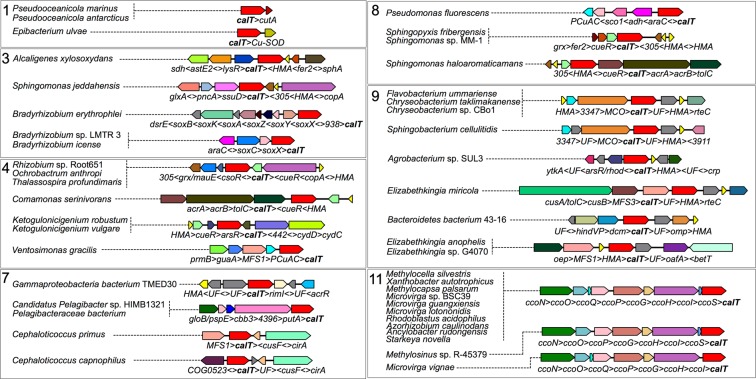
Genomic neighborhoods containing CalT near putative Cu-related proteins. Close proximity between CalT-encoding genes and putative Cu-homeostasis genes was observed for various genomes in clusters 1, 3, 4, 7, 8, 9 and 11. A cartoon for each neighborhood is shown with corresponding genomes (on the left) in which the neighborhood was found. All gene abbreviations are listed in SI Table S4.

Given the experimentally defined role of CcoA as being a Cu transporter^13,14^, we searched within genomic neighborhoods for genes encoding either cuproproteins or other proteins involved in Cu homeostasis. Noticeably, the previously identified group of *Rhizobiales* CalT (cluster 11B) was an example where *calT* could be found located next to the *cbb*_3_-Cox biogenesis genes (*ccoNOQP-ccoGHIS*)^17^. In addition, *calT* homologs were observed next to a gene containing a cytochrome_CBB3 domain (PF13442), similar to subunit III of *cbb*_3_-Cox, in two unclassified *Pelagibacteraceae* bacteria and *Pelagibacter* sp. HIMB1321 (cluster 7), and *Bradyrhizobium* sp. LMTR 3, *Bradyrhizobium icense* and *Bradyrhizobium erythrophlei* (cluster 3 in Fig. 4). In the case of *Bradyrhizobium* spp., this gene putatively encodes SoxX and is found in a putative sulfur-oxidizing gene cluster. This finding is significant as the SoxAX from *Starkeya novella* was shown to contain a mononuclear Cu^2+^ center^23^. Putative Cu chaperones containing a **H**eavy **M**etal **A**ssociated (HMA, PF00403) domain were found in the top 15 neighbors at the family and order levels, with the corresponding CalT proteins being in clusters 3, 4, 7, 8 and 9 (45 of them are shown in Fig. 4). Interestingly, the *O. anthropi* genome contains two *calT* genes in cluster 4; one corresponds to the earlier described *rfnT*^19^, and a paralog is located near a putative Cu chaperone gene. In addition, several clusters contained *copA*, *csoR*, *cueR* or *mco* genes that encode proteins involved in Cu-detoxification (Fig. 4). Out of the 1635 *calT* genes analyzed, only a few were observed proximal to additional genes also encoding Cu-responsive or Cu-homeostasis related proteins, such as CusF (*Cephaloticoccus primus* and *Cephaloticoccus capnophilus*), PCuAC (*Ventosimonas gracilis*), SCO1 (*Pseudomonas tolaasii* and *Pseudomonas fluorescens*), CutA1 (divalent ion tolerance protein, PFAM3091) (*Pseudooceanicola marinus* and *Pseudooceanicola antarcticus*) and Cu/Zn superoxide dismutase (*Epibacterium ulvae*).

### Heterologous expression of RfnT-like CalT proteins in *E. coli*

**Figure 5:**
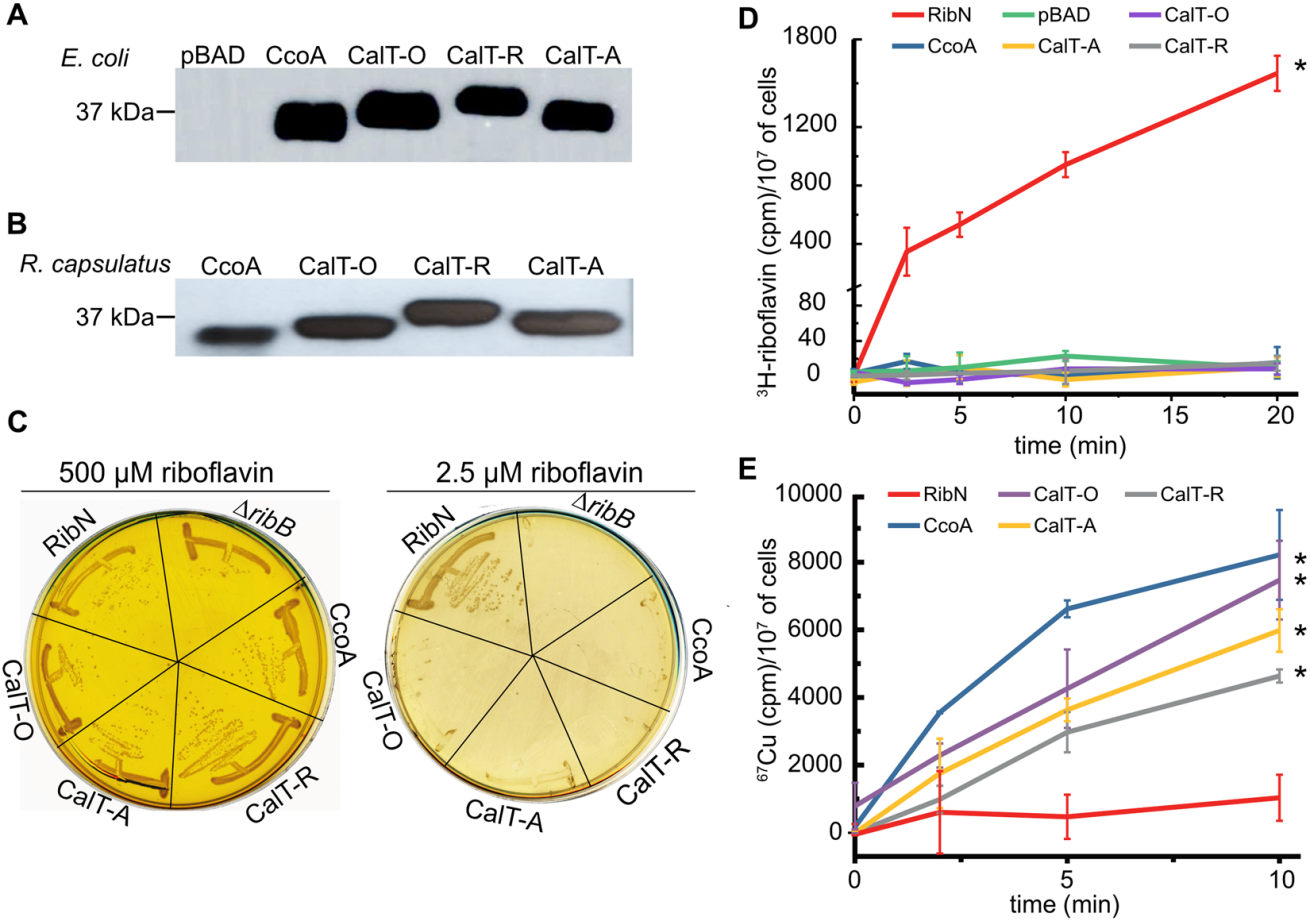
L-Ara inducible production of CcoA and RfnT-like CalT and radioactive ^67^Cu uptake activities of *E. coli* strains expressing them. (**A**) Immunoblot analysis of CcoA from *R. capsulatus* and CalT from *O. anthropi* (CalT-O, formerly called RfnT), *R. palustris* (CalT-R) and *A. tumefaciens* (CalT-A) expressed in *E. coli*. (**B**) Immunoblot analysis of CcoA, CalT-O, CalT-R, CalT-A expressed in *R. capsulatus*. The *E. coli* (LMG194) and *R. capsulatus* ∆*ccoA* (SE8, not shown) strains harboring the empty expression vector pBAD/Myc-His A were used as a negative control and referred to as pBAD. Full-length blots are presented in SI Fig. S2. (**C**) The Δ*ribB* mutants expressing CalT from *O. anthropi* (CalT-O), *R. palustris* (CalT-R) and *A. tumefaciens* (CalT-A), *R. capsulatus* CcoA or *R. leguminosum* RibN were grown at 37 °C on LB plates containing either high (500 µM) (left with yellow background due to high amount of riboflavin) or low (2.5 µM) (right) concentrations of riboflavin. In the presence of 2.5 µM riboflavin, the auxotrophic phenotype of the *E. coli* Δ*ribB* mutant was restored only when cells expressed the *bona fide* riboflavin transporter RibN, but not any one of the CalT proteins. (**D**) Radioactive ^3^H-riboflavin uptake assays using appropriate *E coli* cells (strain LMG194 derivatives, SI Table S2) expressing either CcoA or RibN or the CalT-O, -R or -A. pBAD corresponds to the same cells carrying an empty expression vector (SI Table S2). In each case, the uptake assays were repeated at least three times using at least two independently grown cultures, and statistical analysis was performed using the Student’s *t* test, with *p* < 0.01 as the level of significance between RibN (*) and the other strains. (**E**) ^67^Cu uptake kinetics were performed using *E. coli* strain LMG194 expressing the RfnT-like CalT proteins from *R. capsulatus* CcoA, *O. anthropi* (CalT-O, formerly called RfnT), *R. palustris* (CalT-R) and *A. tumefaciens* (CalT-A), or the riboflavin transporter RibN. All uptake assays were performed at 37 °C and on ice as described in Methods, and in each case the activities detected with cells kept on ice were subtracted from those incubated at 37 °C. Of these corrected values the background activity measured with the *E. coli* strain carrying pBAD/Myc-His (pBAD) were subtracted and plotted in function of time. Each assay was repeated at least three times using multiple independently grown cultures, and statistical analysis was performed using the Student’s *t* test, with *p* < 0.01 as the level of significance between RibN and the other strains (*).

Although in our analysis 190 (out of 1635) CalT members from *Proteobacteria* are found near *ribH*, the phylogenomic analysis alone could not definitively distinguish putative Cu transporters from putative riboflavin transporters. Indeed, phylogenetic clusters that contained *calT* genes clustering with the RBP genes also contained homologs nearby the Cu homeostasis genes (cluster 3, 4 and 11). Thus, to further define the functions of CalT family members, we tested experimentally the ability of CcoA-like and RfnT-like members to transport riboflavin and Cu, respectively. We chose three RfnT-like CalT-encoding genes: *O. anthropi calT* (CalT-O, WP_010660541, previously called RfnT) from cluster 4, *R. palustris calT* (CalT-R, WP_011160333) from cluster 3, and *A. tumefaciens calT* (CalT-A, WP_010971445) from cluster 5. These *calT* homologs were cloned into a L-Ara inducible plasmid, as done earlier with *R. capsulatus* CcoA^16^ (Methods). Upon L-Ara induction, *E. coli* K12 strains (LMG194, Δ*araC*) harboring plasmids carrying *calT-O, calT-R* and *calT-A* (SI Table S2) expressed Myc-tagged CalT-O, CalT-R and CalT-A (M_r_ ranging from 37 to 40 kDa) proteins, as detected by immunoblot analysis of whole cell extracts using anti-Myc antibodies (Fig. 5A). The same *E. coli* strain producing *R. capsulatus* CcoA (running as ~37 kDa)^16^ was used as a control. Similarly, whole cells and chromatophore membranes of a *R. capsulatus* strain lacking CcoA (Δ*ccoA*) harboring appropriate plasmids with *ccoA*, *calT-O, calT-R* and *calT-A* (SI Table S2) also contained comparable amounts of the respective proteins (Fig. 5B), indicating that they were expressed and inserted in the cytoplasmic membrane in these species.

### CalT-O, CalT-R and CalT-A do not complement *E. coliΔribB* mutant for riboflavin auxotrophy

*E. coli* has no known riboflavin transporter, but produces riboflavin via its endogenous RBP, which includes the *ribB* gene encoding the 3,4-dihydroxy-2-butanone-4-phosphate synthase^20,24^. Thus, an *E. coli* Δ*ribB* mutant cannot grow on LB medium unless supplemented with a large amount (500 µM) of riboflavin, which is thought to diffuse passively across the membrane^20^. In contrast, heterologous expression of an efficient riboflavin uptake transporter, such as the *Rhizobium leguminosarum* RibN, enables growth of an *E. coli* Δ*ribB* mutant on LB medium containing low amounts (2.5 µM) of riboflavin^25^.

In order to assess whether heterologous expression of CalT-R, -O and -A could confer riboflavin uptake activity in *E. coli*, plasmids encoding these orthologs were transformed into the *E. coli* Δ*ribB* mutant (BW25141::Δr*ibB*, SI Table S2) using LB plates containing 500 µM riboflavin. These transformants were then tested for growth on LB plates with low concentration of riboflavin (2.5 µM), in the absence and presence (0 to 2%) of L-Ara. A plasmid expressing the *R. leguminosarum* RibN *bona fide* riboflavin importer (SI Table S2) was used as a positive control^25^. Neither the *E. coli* Δ*ribB* mutant, nor its derivatives carrying the *calT- O, -R and -A* genes were able to grow on 2.5 to 10 µM of riboflavin containing plates, irrespective of the presence of L-Ara, unlike those carrying *ribN* (Fig. 5C). As these CalT proteins were expressed in *E. coli* (Fig. 5A), their inability to rescue the growth on low concentration of riboflavin suggested that they could not confer efficient riboflavin uptake to sustain growth of *E. coli*, unlike RibN. Similar results were also obtained with a plasmid (pBK68) carrying *R. capsulatus ccoA*, indicating that CcoA also did not have such uptake activity (Fig. 5C).

During these experiments we observed that the *E. coli* Δ*ribB* mutant (SI Table S2), and its derivatives expressing various CalT yielded spontaneous revertants that regained riboflavin-independent growth ability on LB medium in the absence, or presence of 2.5 µM of riboflavin (Fig. 5C, *e.g*., Δ*ribB* expressing CcoA or CalT-O). These observations suggested that similar events might have occurred during the earlier work with *O. arthropi* gene^19^.

### Neither CcoA nor RfnT-like CalT exhibit riboflavin uptake activity in *E. coli*

*E. coli* cells producing CcoA or RfnT-like CalT were tested for their ability to take up radioactive ^3^H-riboflavin. The data showed that ^3^H-riboflavin was taken up readily by the *E. coli* cells expressing RibN, but not by those expressing the three CalT homologs or CcoA (Fig. 5D). Moreover, in the case of CcoA, which is known to transport Cu, addition of Cu (100 μM) did not affect its inability to take up ^3^H-riboflavin. We concluded that neither CcoA nor the RfnT-like CalT members exhibited any efficient riboflavin uptake activity in *E. coli*, in agreement with their lack of complementation of the *E. coli* Δ*ribB* strain for auxotrophy at low riboflavin amounts, suggesting that the earlier observed growth with *O. anthropi rfnT*^19^ (*i.e*., *calT-O*) might have been due to spontaneous reversion.

### The RfnT-like CalT proteins mediate ^67^Cu uptake activity in *E. coli* cells

The conservation of the CcoA Cu-binding motifs (**M**xxx**M** and **H**xxx**M**) in the RfnT-like CalT proteins led us to investigate whether they could import Cu into *E. coli* cells, like *R. capsulatus* CcoA^15,16^. Time dependent ^67^Cu uptake activities of appropriate strains were measured using whole cells grown in the presence of 0.5% L-Ara. As a control, *E. coli* cells expressing wild-type *R. capsulatus* CcoA exhibited significantly higher amounts of ^67^Cu uptake than the same cells lacking CcoA (*i.e*., CcoA-independent ^67^Cu uptake background, Methods), as reported earlier^16^. Remarkably, *E. coli* cells expressing CalT-O or -R or -A also showed robust ^67^Cu uptake activities, whereas the same *E. coli* (or a Δ*ribB* derivative) cells expressing RibN had no detectable ^67^Cu uptake activity (Fig. 5E). Therefore, we concluded that RfnT-like CalT proteins have Cu, but not riboflavin, uptake activity when expressed in *E. coli*, similar to CcoA. We note that the amounts of ^67^Cu accumulated in *E. coli* cells expressing different CalT proteins were slightly different. This point being out of the scope of this work, the amounts and affinities for Cu of those transporters were not studied further.

### RfnT-like CalT proteins do not complement the *R. capsulatusΔccoA* mutant for *cbb*_3_-Cox defect

**Figure 6:**
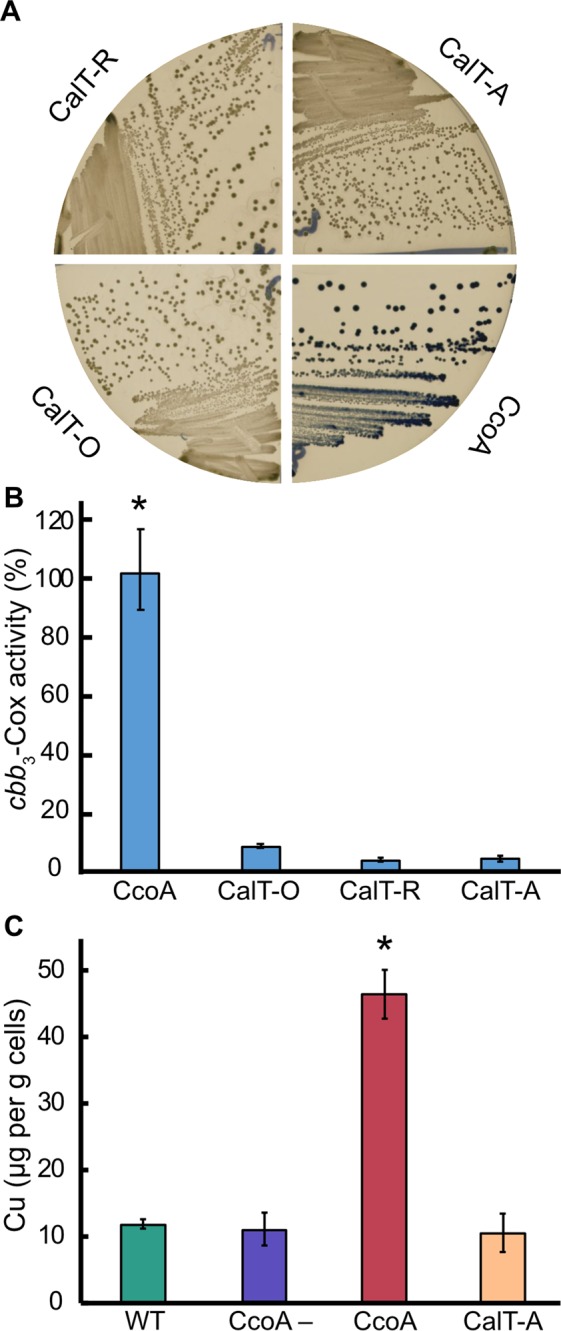
The RfnT-like CalT proteins do not restore the *cbb*_3_-Cox defect of *R. capsulatus* Δ*ccoA* mutant. (**A**) *R. capsulatus* Δ*ccoA* mutant (SE8), the Δ*ccoA* mutant expressing either *R. capsulatus* CcoA (positive control) or the CalT from *O. anthropi* (CalT-O), *R. palustris* (CalT-R) or *A. tumefaciens* (CalT-A) were stained using the NADI procedure (Methods), which detects *cbb*_3_-Cox activity in *R. capsulatus* colonies via the development of a blue color. Note that *cbb*_3_-Cox activity was detected only in the presence of CcoA, and not the CalT-O, -R or -A proteins. (**B**) *In vitro cbb*_3_-Cox activities in chromotophore membranes of *R. capsulatus* SE8 strain expressing either CcoA or CalT-O, -R or -A. Cox assays were initiated by adding 30–150 μg of DDM-dispersed chromatophore membrane proteins to the assay buffer containing 0.1% DDM and 50 μM of reduced cyt *c*. Decrease in absorbance at 550 nm resulting from oxidation of cyt *c* was recorded. Addition of 0.1 mM KCN stopped immediately cyt *c* oxidation confirming the specificity of the *cbb*_3_-Cox activity. The *cbb*_3_-Cox activity determined for SE8/CcoA strain was 462 nmoles of cyt *c* oxidized/min/mg protein and set as 100%. Note that the *R. capsulatus* SE8 strains expressing CalT-O, -R or -A proteins have no *cbb*_3_-Cox activity. Mean values of at least three independent measurements with corresponding error bars are shown, and statistical analysis was performed using the Student’s *t* test, with *p* < 0.01 as the level of significance between CcoA (*) and the other strains. (**C**) Whole-cell Cu content measured by ICP-MS for wild-type *R. capsulatus* (WT), CcoA^−^, (*ΔccoA*) or a CcoA^–^ strain expressing either CcoA or CalT-A. Each bar represents the mean values of four individually digested and independently measured samples from each strain and statistical analysis was performed using the Student’s *t* test, with *p* < 0.01 as the level of significance between CcoA (*) and the other strains.

Considering that CcoA is a Cu importer required for *cbb*_3_-Cox biogenesis in *R. capsulatus*^14,15^ and *R. sphaeroides*^17^, and that the RfnT-like CalT proteins can also import Cu, their ability to complement the *R. capsulatus* Δ*ccoA* mutant for its *cbb*_3_-Cox biogenesis defect was tested. Appropriate plasmids expressing CalT-O or -R or -A were conjugated into a *R. capsulatus* strain lacking CcoA. In parallel, a similar plasmid (pBK69) expressing wild-type *R. capsulatus* CcoA was used as a control (SI Table S2). The trans-conjugants were first tested for the presence of *cbb*_3_-Cox activity using the Nadi staining procedure (Cox activity dependent conversion of α-naphthol to indigo blue^26^). Colonies containing CcoA turned blue (*i.e*., Nadi^+^ phenotype) immediately (<30 sec), while those with the RfnT-like CalT proteins remained unstained upon longer (>10 min) exposure times (Fig. 6A). In addition, supplementation of the growth medium with 1 to 500 nM Cu, to increase Cu availability (in case of the lower uptake activities, or Cu affinities of CalT’s tested) was not efficient. Unfortunately, use of higher amounts of Cu supplementation was not informative because of the phenotypic suppression of a Δ*ccoA* mutant for *cbb*_3_-Cox activity caused by μM amounts of external Cu^13,15^. However, immunoblot analyses of membrane preparations from the trans-conjugants using anti-myc antibodies showed that they all contained membrane-bound RfnT-like CalT proteins at levels comparable to those of CcoA (Fig. 5B). These findings suggested that, although produced and inserted into the membrane, the RfnT-like CalT proteins were unable to yield any active *cbb*_3_-Cox. Indeed, the trans-conjugants expressing CalT-O or -R or -A had very low levels of *cbb*_3_-Cox activity (~3–5%) compared with the *R. capsulatus* Δ*ccoA* complemented with CcoA (100%) (Fig. 6B). Moreover, determination of the total cellular amounts of Cu associated with cells expressing CalT-A showed no accumulation of cellular Cu, unlike those containing CcoA (Fig. 6C) (see also SI Fig. S3 for the metal contents of these cells), suggesting that it was inactive in *R. capsulatus* membranes. Overall data showed that although the RfnT-like CalT proteins exhibited Cu uptake activity in *E. coli* cells, they were unable to complement a *R. capsulatus* strain lacking CcoA for *cbb*_3_-Cox biogenesis.

## Discussion

During our previous comparative genomic study of CcoA required for *cbb*_3_-Cox biogenesis^13–16^ in *R. capsulatus* and *R. sphaeroides*^17^ we noticed that a subgroup of the CcoA homologs (**C**co**A**-**l**ike **t**ransporters or CalT) included the RfnT proteins previously predicted to transport riboflavin^18–20^. Moreover, the conserved (**M**xxx**M** and **H**xxx**M**) motifs of CcoA, which are associated with Cu import and *cbb*_3_-Cox biogenesis^16,27^, were also conserved in this subgroup. This similarity led us to further investigate this subfamily in order to probe whether the different members of the CalT family could transport different substrates such as Cu or riboflavin. We first divided the CalT family into 11 clusters based on the phylogenomic and genomic context analyses. While CcoA from *R. capsulatus* and *R. sphaeroides* belongs to a distinct cluster of proteins (cluster 1) shared with orthologs from other *Rhodobacteraceae*, we were unable to make a clear phylogenetic distinction between putative Cu transporters and putative riboflavin transporters. In the same protein cluster (*e.g*., clusters 3 and 4) we found *calT* genes that were located proximal to HMA-domain containing Cu chaperones involved in Cu response or detoxification, in support of a Cu-related function, whereas in other genomes their orthologs were next to RBP gene clusters. Thus, to establish the substrate specificity of different CalT subfamilies with respect to Cu and riboflavin we used an empirical approach. Three RfnT-like CalT proteins from three *cbb*_3_-Cox encoding proteobacterial species were introduced into appropriate *E. coli* and *R. capsulatus* mutants. Protein expression, phenotypic complementation and radiolabeled Cu and riboflavin uptake kinetics data showed that CalT-O, -R and -A from *O. anthropi* (cluster 4), *R. palustris* (cluster 3) and *A. tumefaciens* (cluster 5), respectively, were MFS-type Cu transporters just like *R. capsulatus* CcoA, and not efficient riboflavin transporters. Conceivably, currently unknown link(s) between Cu and riboflavin might exist, and these proteins may transport Cu and/or riboflavin at much higher concentrations or under specific conditions that are different than those used here. In any event, our findings validated the conservation of the **M**xxx**M** and **H**xxx**M** motifs in these CalT subfamily members, and suggested that this motif may be a good predictor of Cu importers among the MFS transporters. Currently, this point is further pursued using appropriate strains and species.

Most bacteria have an active RBP and are able to synthesize riboflavin *de novo*^20,24^, yet some species can also take up riboflavin from their environment via specific riboflavin uptake transporters^20^. Several such transporters have been described, and among them the energy coupling factor (ECF)-type RibU^28,29^, PnuX/RibM^30–32^, and RibN^19,25^ have been shown to transport riboflavin or its derivatives, whereas some others (*e.g*., ImpX and RibXY) are less studied. With the exception of the well characterized ECF-type RibU^29^, very little is known about the structural properties of bacterial riboflavin transporters and the specific motifs involved in substrate binding. Initially, *rfnT* was proposed to encode another riboflavin transporter based on its physical proximity to the RBP genes in *Rhizobiales* genomes^18,19^. However, neither the expression of CalT-O in the Δ*ribB* mutant, nor an ability to take up riboflavin was examined^19^. During our analyses, we found that the *E. coli* Δ*ribB* mutant (BW25141::Δ*ribB*) used in previous studies reverted spontaneously to riboflavin protrophy. Similarly, the Δ*ribB* derivatives expressing CalT-O, -R and -A yielded riboflavin prototrophic revertants, raising the issue of whether the RfnT-like CalT proteins were efficient riboflavin transporters. Indeed, ^3^H-riboflavin uptake experiments showed that cells harboring these proteins (and even CcoA) were unable to take up riboflavin, unlike a *bone fid*e riboflavin transporter (e.g., *R. leguminosarum* RibN^25^). Instead, these transporters also exhibited Cu-transport activity in *E. coli* like *R. capsulatus* CcoA.

The unusual association of some *calT* subfamilies with RBP genes might suggest a possible, but currently unknown role for riboflavin in Cu homeostasis or Cu in riboflavin biosynthesis, or even cytochrome biogenesis in bacteria. Notably, some *calT* genes located in neighborhood N3 were found to be associated with a gene encoding a putative chaperone of ubiquinol-cytochrome *c* oxidoreductase. Moreover, a recent work using transcriptomics suggested that RibN-imported riboflavin might be involved in *c*-type cytochrome biogenesis in *Vibrio cholerae*^21^.

An unexpected finding was the inability of the RfnT-like CalT proteins to complement the *cbb*_3_-Cox defect of a *R. capsulatus* mutant lacking CcoA. Considering the successful heterologous production and membrane localization of CalT-O, -R and -A in *R. capsulatus*, and their Cu uptake activities seen in *E. coli*, the basis of this observation remains unclear. A possibility is that the RfnT-like CalT subfamily members might be inactive for unknown reason(s) for Cu uptake in *R. capsulatus* despite their competence in *E. coli*. The ICP-MS data suggested that *R. capsulatus* cells producing CalT-A do not accumulate Cu unlike those containing CcoA. A different possibility is that the Cu uptake and delivery pathways during *cbb*_3_-Cox biogenesis via the CalT family members might be species-specific. If so, these proteins (or the chemical nature of Cu cargo) might be incompatible to interact with their heterologous partner(s) to convey Cu to its ultimate destination, rendering them non-interchangeable for *cbb*_3_-Cox biogenesis. Similar diversity occurs with cytoplasmic Cu chaperones in lower eukaryotes^33^. Ongoing work aiming at inactivating a RfnT-like CalT member (*i.e*., CalT-A) in a genetically tractable species like *A. tumefaciens*, and defining its effect(s) on *cbb*_3_-Cox biogenesis and Cu transport might shed further light to some of these issues. Moreover, the role of CalT in the provision of Cu to other cuproproteins also remains a possibility as not all CalT-encoding genomes encode a *cbb*_3_-Cox.

Finally, the biogenesis of *cbb*_3_-Cox is a complex process that is not yet fully understood^12,27^. It involves an increasing number of Cu chaperones and transporters, including SenC (PrrC/Sco homolog) and PccA (PCuAC homolog)^34–36^ that work collaboratively^37^ with the dedicated P_1B_-type transporter CcoI (also known as CtpA/CopA2)^38–40^. The spatial and temporal order(s) with which these Cu chaperones handle Cu, and interact with each other, is only now emerging^27^. In the absence of a three-dimensional structure for a CalT member, it is difficult to speculate about the amino acid residues that might be responsible for the observed differences. Nonetheless, sequence alignments show salient differences located around the cytoplasmic and periplasmic loops between the TM6 - TM7 and TM11 - TM12 of CalT members, respectively (SI Fig. S4). The occurrence of amino acid residues that are conserved in the CcoA and not in the RfnT-like CalT subfamilies, and *vice versa*, might be important in defining their specificity.

In summary, this study further defined the extended family of CalT in *Proteobacteria* and demonstrated that the RfnT-like CalT subfamily members are not riboflavin transporters, but they are rather *bona fide* Cu importer members of the Cu Uptake Porter family of TCDB^1^. Moreover, the occurrence of the conserved **M**xxx**M** and **H**xxx**M** motifs among this family appears to be a reliable predictor of Cu import activity. Whether all members of the CalT family exclusively provide Cu to the Cu_B_ center of *cbb*_3_-Cox, or to other cuproproteins as well remains to be seen.

## Methods

### Bacterial strains and growth conditions

The bacterial strains and plasmids used in this study are listed in SI Table S2. Standard molecular biology techniques were used^41^. *E. coli* strains were grown in LB medium at 37 °C supplemented as needed with ampicillin (Amp), chloramphenicol (Cm), tetracycline (Tet) and kanamycin (Km) at final concentrations of 100, 30, 12.5 and 50 μg/mL, respectively^42,43^. *E. coli* Δ*ribB* strains were grown in the presence of 500 μM riboflavin, because they are unable to grow at lower concentrations (*e.g*., 2.5 μM) unless they express a functional heterologous riboflavin transporter (*e.g*., RibN)^25^. Complementation of this auxotrophic growth phenotype of the Δ*ribB* strain was used to assess the ability of a gene product to transport riboflavin upon heterologous expression. *E. coli* strains containing pBAD/Myc-His A plasmid derivatives were grown overnight in LB medium with 0.5% L-arabinose (L-Ara) to express L-Ara-inducible genes. The *R. capsulatus* SE8 (Δ*ccoA*) strain derivatives were grown in enriched medium (MPYE) at 35 °C supplemented with 2.5 μg/mL Tet. The L-Ara-inducible pBAD-pRK415 plasmid derivatives were conjugated into *R. capsulatus* by tri-parental mating using the helper plasmid pRK2013^42,44^ and cells were grown overnight in the presence of L-Ara (0.5% to 2% as needed)^16^.

### Construction of the expression plasmids

*R. palustris calT* gene (*calT-*R) was amplified using primers RPA-F and RPA-R (SI Table S3), and the resulting 1248 bp PCR fragment was digested with *Hind*III and *Kpn*I and cloned into pBAD/Myc-His A vector, yielding plasmid pYZ02 encoding a C-terminally Myc-His-fused CalT-R (SI Table S2). Similarly, a 1224 bp PCR fragment containing the *calT* gene from *A. tumefaciens* (*calT-*A) was amplified using primers Atu-F and Atu-R, and cloned into pBAD/Myc-His A as above, yielding plasmid pYZ03 with a Myc-His tagged CalT-A. The *calT* gene (previously called *rfn*T^18^) from *O. anthropi* (*calT-*O) was amplified using primers OanT-F and OanT-R (SI Table S3) resulting in a 1197 bp PCR fragment that was cloned into pBAD/Myc-His A digested with *EcoR*I and *Kpn*I, yielding the plasmid pYZ09 with a Myc-His tagged CalT-O (SI Table S2). Plasmids pYZ02 and pYZ09 were digested with *Nsi*I, and ligated into the broad-host-range vector pRK415 digested with *PstI* (compatible cohesive ends with *NsiI*), yielding pYZ07 and pYZ11, respectively (SI Table S2). As the wild-type *calT-*A contains an internal *Nsi*I (ATGCAT) site, the adenine of the *Nsi*I site was replaced by a cytosine (ATGCCT) using the Q5 Site-Directed Mutagenesis Kit (NEB, Beverly, MA). Plasmid pYZ03 and primers AtuN-F and AtuN-R were used, yielding plasmid pYZ08 that was digested with *Nsi*I and ligated into pRK415, digested with *Pst*I, yielding plasmid pYZ13 (SI Table S2).

### Whole cell lysates and chromatophore membrane preparation, SDS-PAGE and immunoblots

For whole-cell lysates, *E. coli* (1.5 mL) and *R. capsulatus* (10 mL) cultures were grown overnight in the presence of 0.5% L-Ara^16^. Cells were collected by centrifugation, washed and resuspended in CelLytic™ B Cell Lysis Reagent (Sigma-Aldrich, Saint Louis, MO) (200 μL) supplemented with lysozyme (20 μg/mL), DNAse (10 μg/ mL) and phenylmethylsulfonyl fluoride (PMSF, 200 μM), and incubated at room temperature for 15 min with shaking. Lysates were supplemented with EDTA (4 μM) and cleared by centrifugation at 19000 × *g* for 15 min. Proteins (30 μg of cell lysates or 40–60 μg of chromatophore membranes) were separated using 12% Tris-Glycine SDS-PAGE and transferred to Immobilon-P PVDF membranes. For chromatophore membrane preparations, *R. capsulatus* strains were grown in 1 L cultures. Cells were collected and resuspended in 25 mM Tris pH 7.5, 150 mM NaCl buffer, 200 μM PMSF (Cox assay buffer) and intracytoplasmic membrane vesicles were prepared as done earlier^45^. Protein concentrations were determined using the bicinchoninic acid assay (Sigma Inc.; procedure TPRO-562). Immunoblot analysis to detect the presence of the c-Myc epitope using either *E. coli* or *R. capsulatus* cell extracts or chromatophore membrane proteins (*R. capsulatus*) was done as in^16^. The presence of the CcoA or CalT in cell lysates of *E. coli*, and in the membrane fraction of *R. capsulatus* was confirmed by immuno-detection using anti-Myc monoclonal antibody and horseradish peroxidase conjugated anti-mouse IgG. Signal was detected using the Supersignal West Pico chemiluminescence substrate.

### *In vivo* and *in vitro* cbb_3_-Cox activity

Tet^R^ derivatives of *R. capsulatus* SE8 (Δ*ccoA*) containing plasmids pYZ07, pYZ11, pYZ13 and pBK69 (SI Table S2) were purified on appropriate MPYE plates under respiratory growth conditions, and their *cbb*_3_-Cox activities visualized qualitatively with the Nadi staining procedure^26^. Staining of the colonies was done as previously described^17^. The *cbb*_3_-Cox activities were measured by monitoring oxidation of reduced horse heart cyt *c* (Sigma Inc.) using chromatophore membranes according to^17,46^.

### Whole cells ^67^Cu and ^3^H-riboflavin uptake assays

The Cu uptake assays were performed according to^15^. Radioactive ^67^Cu (half-life of ~62 hours) was obtained from the DOE-Brookhaven National Laboratory (NY). *E. coli* strain LMG194 containing the pBAD/Myc-His derivatives encoding *R. capsulatus ccoA* (pBK68) or various *calT* (pYZ02, pYZ03 and pYZ09), or *R. leguminasorum* riboflavin transporter RibN (pGRibN)^25^ (SI Table S2) were grown in 10 mL of LB supplemented with 0.5% L-Ara until an OD_600_ of 0.5. Similarly, the *E. coli* strains BW25141::Δ*ribB* (Δ*ribB* derivative of BW25141)^19^ and BW25141::Δ*ribB*/pGRibN were grown in the presence of appropriate amounts of riboflavin as control strains. Cells were collected, washed with 50 mM sodium citrate, pH 6.5, 5% glucose buffer (uptake assay buffer) and re-suspended in 1 mL of the uptake assay buffer. Optical density at 600 nm was determined. For each assay, a total of 7.5 × 10^8^ cells per 500 µL of total assay mixture (1.0 A_600_ = 5 × 10^8^ cells/mL) were used. Cells were incubated for 10 min either at 35 °C or on ice, before each assay. Cu uptake was initiated by addition of 10^6^ cpm of ^67^Cu (determined immediately before use) to the cell suspension. At each time point (0, 1, 2, 5, and 10 min), aliquots of 50 µL of assay mixture were collected and combined with 50 µl of CuCl_2_ (1 mM) and 50 µL of EDTA (50 mM, pH 6.5) to stop the uptake activity, and stored on ice. The aliquots were then centrifuged, and cells washed twice with 100 µL of ice-cold EDTA (50 mM, pH 6.5) solution. Cells were re-suspended in 1 mL of scintillation liquid (Bio-Safe II, RPI, Mt. Prospect, IL) and counted using a scintillation counter with a wide-open window. Background uptake activities determined using cell mixtures kept on ice during the assays were subtracted from those obtained at 35 °C, and plotted in function of time.

For ^3^H-riboflavin (Moravek Inc., Brea, CA) uptake assays, *E. coli* strains were grown to an OD_600_ of 0.4–0.6, washed with LB medium and re-suspended in LB medium to a final OD_600_ of 12. A total of 7.5 × 10^8^ cells were diluted with uptake assay buffer to a final volume of 500 µL. Assay mixtures were pre-incubated either at 37 °C or kept on ice for 10 min before initiating the assay by addition of 2.5 μM riboflavin containing 2 μCi of ^3^H-riboflavin. At each time point (0, 2, 5, 10, and 20 min), an aliquot of 50 µL was taken and mixed with 50 µL of ice cold stopping solution (100 μM non-radioactive riboflavin in LB medium) and stored on ice. Cells were then pelleted, washed with 500 µL of stopping solution and re-suspended in 1 mL of scintillation liquid, and counted using a scintillation counter (Tri-Carb 2900 TR, Perkin Elmer).

### Determination of total cellular Cu contents using ICP-MS

Samples for determination of total cellular Cu contents were prepared as described earlier^15^. Briefly, *R. capsulatus* strains were grown by respiration in 1 L of enriched MPYE medium prepared with metal-free water (stirred at room temperature with Chelex100 at a concentration of 5 g/L for 1 hour) to an OD_630_ of 0.8–0.9. Cells were harvested by centrifugation and washed three times with metal-free 20 mM Tris-HCl pH 8.0 and once with ice cold metal free water. Cell pellets were lyophilized to complete dryness. A total of 50 mg of dry cell powder per sample was digested in 1 ml trace-metal grade nitric acid (Sigma) at 65 °C. To obtain a corresponding blank, the volume of the cell powder was replaced by milli-Q grade water (ultrapure) and treated the same as the samples. The digested samples were then diluted with milli-Q grade water to a final concentration of 1 mg/ml cell powder. Total metal content was measured by ICP-MS (Nexion 350D, Perkin Elmer equipped with an Element Scientific prepFAST M5 autosampler) using quadruplicate digested samples for each strain.

### Comparative genomic and phylogenetic analyses

The protein similarity network was constructed using the EFI-EST tool (http://efi.igb.illinois.edu/efi-est/)^47^ with an alignment score of 75. CalT proteins that were not connected to the main network hub were deleted and not included in further analyses. A full list of identified CalT members was published previously^17^, and the sequences used in this study are available in SI Table S1. The network was visualized with the yFiles organic layout provided with the Cytoscape software (http://www.cytoscape.org)^48^. The nodes in the network were colored either by taxonomy as provided by the UniProt database^49^, by cluster as determined by the phylogenetic analysis, or by the presence of proteins containing CcoN (IPR004677), CcoO (IPR003468) and CcoP (IPR004678 or IPR032858) as determined with the Interpro database^50^. The phylogenetic analysis was performed using NCBI’s COBALT^51^ for sequence alignment and IQ Tree^52^ as implemented on the CIPRES web portal^53^ with 1000 bootstrap replicates^54^. In addition to the sequences found in the network, 12 *Rhizobiales* CalT sequences, which are encoded by genes found near the *cbb*_3_-Cox biogenesis cluster were added to the phylogenetic analysis. Before tree building, the multiple-sequence alignment was edited to remove positions with a quality score less than 826^55^ and those sequences that did not contain the **M**xxx**M** and **H**xxx**M** motifs. Sequence logos were built with Skylign^56^ using the same multiple-sequence alignment used for the phylogenetic analysis.

Gene neighborhoods (a window of three genes upstream and downstream of each gene encoding a CalT protein from the similarity network) were retrieved using the EFI-GNT tool (https://efi.igb.illinois.edu/efi-gnt/). At the genus level, we identified 605 protein family (Pfam) domains or domain fusions (referred to as neighbors) that were seen in at least two different genera. We ranked these domains by number of genera and set a threshold at 30 individual genera, which resulted in 19 neighboring PFam domains. Of these, transcription factors, PF07690 (MFS_1) and PF00005 (ABC_tran) were excluded from further analysis because they are particularly large multi-functional families. The remaining 17 neighboring PFam domains could be collapsed into three main neighborhoods (SI Table S1).

### Statistics analysis

The data are presented as means ± S.D, and statistical analysis was performed using the Student’s *t* test, with *p* < 0.01 as the level of significance and indicated in the figure legends.

## Supplementary information

Supplementary information

Supplementary Table S1

## References

[1] Saier JMH (2016). The Transporter Classification Database (TCDB): Recent Advances. Nucleic Acids Research.

[2] Marger MD, Saier MH (1993). A Major Superfamily of Transmembrane Facilitators that Catalyse Uniport, Symport and Antiport. Trends in Biochemical Sciences.

[3] Pao SS, Paulsen IT, Saier MH (1998). Major Facilitator Superfamily. Microbiology and Molecular Biology Reviews.

[4] Zhang XC, Zhao Y, Heng J, Jiang D (2015). Energy Coupling Mechanisms of MFS Transporters. Protein Science.

[5] Newstead S (2011). Crystal Structure of a Prokaryotic Homologue of the Mammalian Oligopeptide–Proton Symporters, PepT1 and PepT2. EMBO J.

[6] Abramson J (2003). Structure and Mechanism of the Lactose Permease of *Escherichia coli*. Science.

[7] Huang Y, Lemieux MJ, Song J, Auer M, Wang D-N (2003). Structure and Mechanism of the Glycerol-3-Phosphate Transporter from *Escherichia coli*. Science.

[8] Deng D (2014). Crystal structure of the human glucose transporter GLUT1. Nature.

[9] Law CJ, Maloney PC, Wang D-N (2008). Ins and Outs of Major Facilitator Superfamily Antiporters. Annual review of microbiology.

[10] Yan N (2013). Structural Advances for the Major Facilitator Superfamily (MFS) Transporters. Trends in Biochemical Sciences.

[11] Quistgaard EM, Löw C, Guettou F, Nordlund P (2016). Understanding Transport by the Major Facilitator Superfamily (MFS): Structures Pave the Way. Nature Reviews Molecular Cell Biology.

[12] Ekici S, Pawlik G, Lohmeyer E, Koch H-G, Daldal F (2012). Biogenesis of *cbb*_3_-type cytochrome *c* oxidase in *Rhodobacter capsulatus*. Biochimica et Biophysica Acta.

[13] Ekici S, Yang H, Koch H-G, Daldal F (2012). Novel Transporter Required for Biogenesis of *cbb*_3_-Type Cytochrome *c* Oxidase in *Rhodobacter capsulatus*. mBio.

[14] Beaudoin J (2013). Copper transport and regulation in *Schizosaccharomyces pombe*. Biochemical Society Transactions.

[15] Ekici S (2014). Intracytoplasmic Copper Homeostasis Controls Cytochrome *c* Oxidase Production. mBio.

[16] Khalfaoui-Hassani B, Verissimo AF, Koch H-G, Daldal F (2016). Uncovering the Transmembrane Metal Binding Site of the Novel Bacterial Major Facilitator Superfamily-Type Copper Importer CcoA. mBio.

[17] Khalfaoui-Hassani B (2018). Widespread Distribution and Functional Specificity of the Copper Importer CcoA: Distinct Cu Uptake Routes for Bacterial Cytochrome c Oxidases. mBio.

[18] Vitreschak AG, Rodionov DA, Mironov AA, Gelfand MS (2002). Regulation of Riboflavin Biosynthesis and Transport Genes in Bacteria by Transcriptional and Translational Attenuation. Nucleic Acids Research.

[19] Gutiérrez-Preciado A (2015). Extensive Identification of Bacterial Riboflavin Transporters and Their Distribution across Bacterial Species. PLoS ONE.

[20] García-Angulo VA (2017). Overlapping Riboflavin Supply Pathways in Bacteria. Critical Reviews in Microbiology.

[21] Sepulveda-Cisternas I, Lozano Aguirre L, Fuentes Flores A, Vasquez Solis de Ovando I, Garcia-Angulo VA (2018). Transcriptomics reveals a cross-modulatory effect between riboflavin and iron and outlines responses to riboflavin biosynthesis and uptake in Vibrio cholerae. Sci Rep.

[22] Overbeek R, Fonstein M, D’Souza M, Pusch GD, Maltsev N (1999). The Use of Gene Clusters to Infer functional Coupling. Proc Natl Acad Sci USA.

[23] Kappler U (2008). SoxAX cytochromes, a new type of heme copper protein involved in bacterial energy generation from sulfur compounds. J Biol Chem.

[24] Fischer M, Bacher A (2005). Biosynthesis of Flavocoenzymes. Nat. Prod. Rep..

[25] García Angulo VA (2013). Identification and Characterization of RibN, a Novel Family of Riboflavin Transporters from *Rhizobium leguminosarum* and Other Proteobacteria. Journal of Bacteriology.

[26] Koch H-G, Hwang O, Daldal F (1998). Isolation and Characterization of *Rhodobacter capsulatus* Mutants Affected in Cytochrome *cbb*_3_ Oxidase Activity. Journal of Bacteriology.

[27] Khalfaoui-Hassani, B. *et al*. In *Cytochrome Complexes: Evolution, Structures, Energy Transduction, and Signaling* (eds William A. Cramer & Toivo Kallas) 527–554 (Springer Netherlands, 2016).

[28] Karpowich NK, Song JM, Cocco N, Wang D-N (2015). ATP Binding Drives Substrate Capture in an ECF Transporter by a Release and Catch Mechanism. Nature Structural & Molecular Biology.

[29] Peng Z, Jiawei W, Yigong SHI (2010). Structure and Mechanism of the S Component of a Bacterial ECF Transporter. Nature (Lond.).

[30] Grill S (2007). Identification and Characterization of two *Streptomyces davawensis* Riboflavin Biosynthesis gene Clusters. Archives of Microbiology.

[31] Hemberger S (2011). RibM from *Streptomyces davawensis* is a Riboflavin/Roseoflavin Transporter and may be Useful for the Optimization of Riboflavin Production Strains. BMC Biotechnology.

[32] Vogl C (2007). Characterization of Riboflavin (Vitamin B(2)) Transport Proteins from Bacillus subtilis and *Corynebacterium glutamicum*. Journal of Bacteriology.

[33] Robinson NJ, Winge DR (2010). Copper metallochaperones. Annu Rev Biochem.

[34] Swem DL, Swem LR, Setterdahl A, Bauer CE (2005). Involvement of SenC in Assembly of Cytochrome c Oxidase in *Rhodobacter capsulatus*. Journal of Bacteriology.

[35] Thompson AK, Gray J, Liu A, Hosler JP (2012). The roles of *Rhodobacter sphaeroides* copper chaperones PCu(A)C and Sco (PrrC) in the assembly of the copper centers of the *aa*_3_-type and the *cbb*_3_-type cytochrome *c* oxidases. Biochimica et Biophysica Acta.

[36] Trasnea P-I (2016). Cooperation Between Two Periplasmic Copper Chaperones is Required for Full Activity of the *cbb*_3_-type Cytochrome c Oxidase and Copper Homeostasis in *Rhodobacter capsulatus*. Molecular Microbiology.

[37] Trasnea, P.-I. *et al*. A Copper Relay System Involving Two Periplasmic Chaperones Drives *cbb*_3_-type Cytochrome *c* Oxidase Biogenesis in *Rhodobacter capsulatus*. *ACS Chem. Biol*., 10.1021/acschembio.8b00293 (2018). 10.1021/acschembio.8b00293 PMC5959785 29613755

[38] Koch H-G, Winterstein C, Saribas AS, Alben JO, Daldal F (2000). Roles of the *ccoGHIS* Gene Products in the Biogenesis of the *cbb*_3_-type Cytochrome *c* Oxidase. Journal of Molecular Biology.

[39] Hassani BK, Astier C, Nitschke W, Ouchane S (2010). CtpA, a Copper-translocating P-type ATPase Involved in the Biogenesis of Multiple Copper-requiring Enzymes. The Journal of Biological Chemistry.

[40] González‐Guerrero M, Raimunda D, Cheng X, Argüello José M (2010). Distinct Functional Roles of Homologous Cu^+^ Efflux ATPases in *Pseudomonas aeruginosa*. Molecular Microbiology.

[41] Sambrook, J. *Molecular Cloning: A Laboratory Manual* (Cold Spring Harbor Laboratory, 2001).

[42] Atta-Asafo-Adjei E, Daldal F (1991). Size of the amino acid side chain at position 158 of cytochrome *b* is critical for an active cytochrome *bc*_1_ complex and for photosynthetic growth of *Rhodobacter capsulatus*. Proceedings of the National Academy of Sciences of the United States of America.

[43] Gray KA, Davidson E, Daldal F (1992). Mutagenesis of methionine-183 drastically affects the physicochemical properties of cytochrome c1 of the bc1 complex of Rhodobacter capsulatus. Biochemistry.

[44] Darrouzet E, Daldal F (2002). Movement of the iron-sulfur subunit beyond the ef loop of cytochrome b is required for multiple turnovers of the bc1 complex but not for single turnover Qo site catalysis. J Biol Chem.

[45] Cooley JW, Ohnishi T, Daldal F (2005). Binding dynamics at the quinone reduction (Qi) site influence the equilibrium interactions of the iron sulfur protein and hydroquinone oxidation (Qo) site of the cytochrome bc1 complex. Biochemistry.

[46] Onder O (2017). Absence of Thiol-Disulfide Oxidoreductase DsbA Impairs cbb3-Type Cytochrome c Oxidase Biogenesis in Rhodobacter capsulatus. Front Microbiol.

[47] Gerlt JA (2015). Enzyme Function Initiative-Enzyme Similarity Tool (EFI-EST): A web tool for generating protein sequence similarity networks. Biochim Biophys Acta.

[48] Shannon P (2003). Cytoscape: a software environment for integrated models of biomolecular interaction networks. Genome Res.

[49] The UniProt C (2017). UniProt: the universal protein knowledgebase. Nucleic Acids Res.

[50] Finn RD (2017). InterPro in 2017-beyond protein family and domain annotations. Nucleic Acids Res.

[51] Papadopoulos JS, Agarwala R (2007). COBALT: constraint-based alignment tool for multiple protein sequences. Bioinformatics.

[52] Nguyen LT, Schmidt HA, von Haeseler A, Minh BQ (2015). IQ-TREE: a fast and effective stochastic algorithm for estimating maximum-likelihood phylogenies. Mol Biol Evol.

[53] Miller, M. A., Pfeiffer, W. & Schwartz, T. Creating the CIPRES Science Gateway for inference of large phylogenetic trees. *Proceedings of the Gateway Computing Environments Workshop (GEL)*, 1–8 (2010).

[54] Hoang DT, Chernomor O, von Haeseler A, Minh BQ, Vinh LS (2018). UFBoot2: Improving the Ultrafast Bootstrap Approximation. Mol Biol Evol.

[55] Waterhouse AM, Procter JB, Martin DM, Clamp M, Barton GJ (2009). Jalview Version 2–a multiple sequence alignment editor and analysis workbench. Bioinformatics.

[56] Wheeler TJ, Clements J, Finn RD (2014). Skylign: a tool for creating informative, interactive logos representing sequence alignments and profile hidden Markov models. BMC Bioinformatics.

