# Supplementary material for: Cu Transport by the Extended Family of CcoA-like Transporters (CalT) in Proteobacteria
Source: Sci Rep. 2019 Feb 4;9:1208. doi: 10.1038/s41598-018-37988-4 (PMC6362234; doi:10.1038/s41598-018-37988-4)
Supplement: Supplementary file 1 — Supplementary information [file 41598_2018_37988_MOESM1_ESM.pdf]

SUPPLEMENTARY INFORMATION

Cu Transport by the Extended Family of CcoA-like Transporters (CalT) in Proteobacteria

Yang Zhang[1#&], Crysten E. Blaby-Haas[2#], Stefan Steimle[1], Andreia F. Verissimo[1+], Victor A. Garcia-Angulo[3], Hans-Georg Koch[4], Fevzi Daldal[1] and Bahia Khalfaoui-Hassani[1*]

[1]Department of Biology, University of Pennsylvania, Philadelphia, PA 19104, USA; [2]Biology Department, Brookhaven National Laboratory, Upton, NY 11973, USA; [3]Microbiology and Mycology Department, Insituto de Ciencias Biomédicas, University of Chile, Santiago, Chile; Institut für Biochemie und Molekularbiologie, ZBMZ, Faculty of Medicine, Stefan-Meier-Strasse 17, Albert-Ludwigs-Universität Freiburg, 79104 Freiburg, Germany.

Keywords: MFS-type transporter, Riboflavin transport, Copper transport, *cbb*$_3$ cytochrome *c* oxidase, CcoA and RfnT.

[#]these authors contributed equally and are both first authors

Present addresses: [*]IPREM, UMR CNRS 5254, and Université de Pau et des Pays de l'Adour, BP1155 Pau, France; [&]School of Life Science, Xiamen University, Xiamen 361102, China; [+]bioMT-Institute for Biomolecular Targeting, Geisel School of Medicine at Dartmouth, Hanover, NH 03755, USA

Corresponding Author: Fevzi Daldal fdaldal@sas.upenn.edu ; phone: 215-898-4394

Figure Legends

Fig S1. CcoA-like transporter (CalT) Protein Similarity Network. A. Nodes are colored by taxonomic classes as indicated in Fig. 1 of the Manuscript. The locations of nodes representing proteins examined in this study are indicated with red arrows. **B.** Nodes are colored by the presence of *cbb*$_3$-Cox genes in the corresponding genome as indicated. For the metagenomics samples where it was not possible to determine whether two genes belong to the same genome, corresponding CalT nodes are not colored. Note that not all *rfnT*-like *calT* genes are next to RBP clusters, and not all genomes contain the *cbb*$_3$-Cox genes.

Fig S2. Phylogenetic tree of CALT proteins found near the RBP gene clusters. A. Phylogenetic tree of those family members that are encoded by genes found near the RBP gene clusters Background coloring denotes shared taxonomy (either *Rhizobiales*, *Rhodospirillaes* or *Rhodobacterales*), while edge coloring denotes the phylogenetic cluster from **Fig. 2A** to which they belong (provided in legend). **B.** Cartoons representing the different genomic neighborhoods observed for each protein cluster shown in **A**. For the gene abbreviations see Table S

Fig S3. Metal contents of CcoA and RfnT-like CalT-A containing cells. Metal contents of appropriate samples were determined by ICP-MS using quadruplicate samples prepared as described in Methods. Strains used (WT (*R. capsulatus*), CcoA-, CcoA+/CcoA- and CalT+/CcoA-) are described in **Table S2**. Statistical analysis was performed using the Student's t test, with $p < 0.01$ as the level of significance between CcoA (*) and the other strains.

Fig S4. Comparison of CcoA to RfnT-like CalT proteins. A schematic of the 12 TM of CcoA and RfnT-like CalT proteins and their cytoplasmic (in) and periplasmic (out) loops are shown. The cytoplasmic loop between the TM6 -TM7, and the periplasmic loop between the TM11- TM12 are least conserved. Selected (M, C and H) amino acid residues that are conserved in CcoA, but not in the RfnT-like CalT members, and *vice versa* are highlighted with a black-outlined box, while those same residues conserved in all four proteins are highlighted with a blue box. Blue diamonds in TM7 and TM8 highlight the position of the putative Cu-binding residues.

Fig. S5 Full-length SDS-PAGE/immunoblots of data shown in Fig. 5A and B.

Table S1.xlsx Datafile for Protein Similarity Network.

Table S2. Strains and plasmids used in this study.

Table S3. Oligonucleotides used in this study.

Table S4. List of gene abbreviations used in the figures of this study.

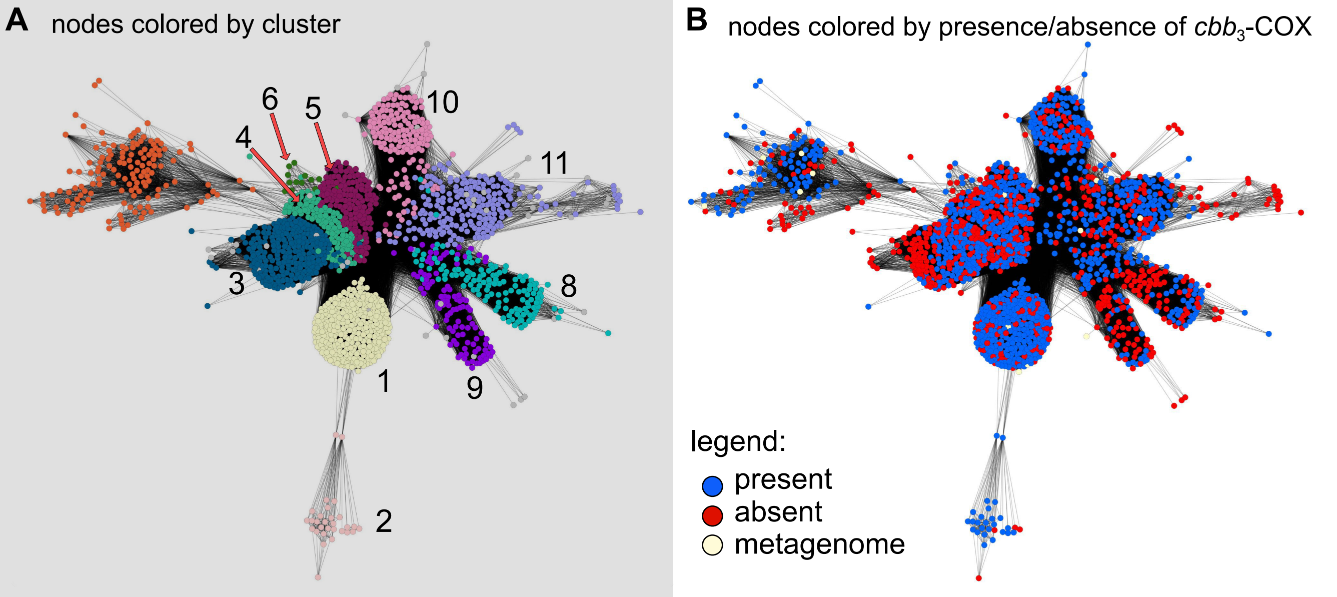
A
nodes colored by cluster
1
2
3
4
5
6
8
9
10
11
B
nodes colored by presence/absence of cbb3-COX
legend:
present
absent
metagenome

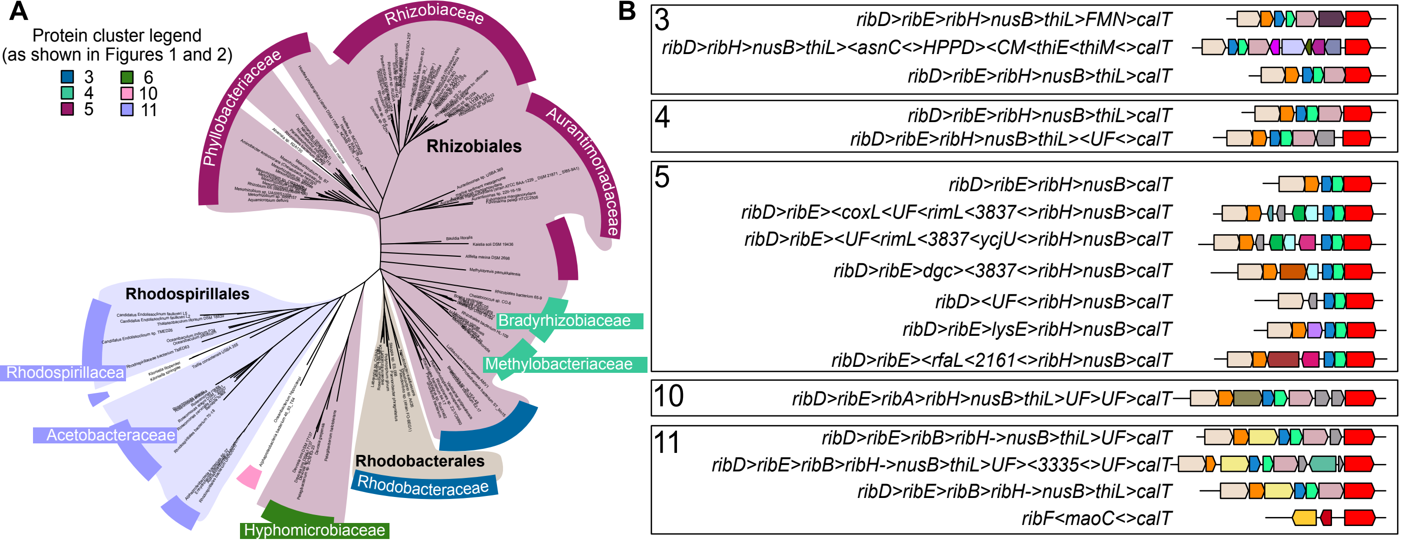
A
Protein cluster legend
(as shown in Figures 1 and 2)
3
4
5
6
10
11
Rhizobiaceae
Phyllobacteriaceae
Rhizobiales
Aurantimonadaceae
Bradyrhizobiaceae
Methylobacteriaceae
Rhodospirillales
Rhodospirillacea
Acetobacteraceae
Hyphomicrobiaceae
Rhodobacterales
Rhodobacteraceae
B
3
ribD>ribE>ribH>nusB>thiL>FMN>calT
ribD>ribH>nusB>thiL><asnC<>HPPD><CM<thiE<thiM<>calT
ribD>ribE>ribH>nusB>thiL>calT
4
ribD>ribE>ribH>nusB>thiL>calT
ribD>ribE>ribH>nusB>thiL><UF<>calT
5
ribD>ribE>ribH>nusB>calT
ribD>ribE><coxL<UF<rimL<3837<>ribH>nusB>calT
ribD>ribE><UF<rimL<3837<ycjU<>ribH>nusB>calT
ribD>ribE>dgc><3837<>ribH>nusB>calT
ribD><UF<>ribH>nusB>calT
ribD>ribE>lysE>ribH>nusB>calT
ribD>ribE><rfaL<2161<>ribH>nusB>calT
10
ribD>ribE>ribA>ribH>nusB>thiL>UF>UF>calT
11
ribD>ribE>ribB>ribH->nusB>thiL>UF>calT
ribD>ribE>ribB>ribH->nusB>thiL>UF><3335<>UF>calT
ribD>ribE>ribB>ribH->nusB>thiL>calT
ribF<maoC<>calT

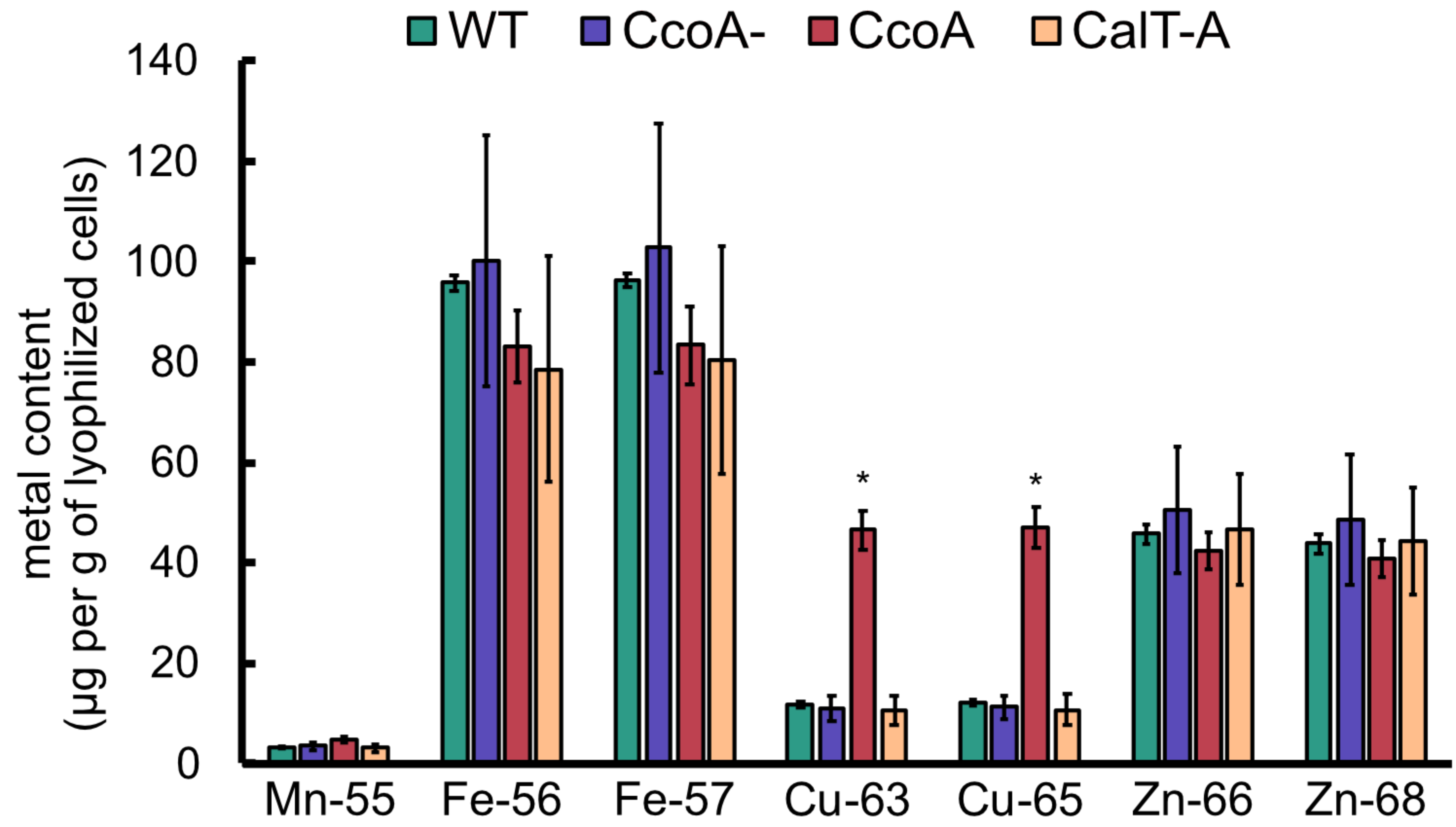

WT
CcoA-
CcoA
CalT-A
metal content
(µg per g of lyophilized cells)
140
120
100
80
60
40
20
0
*
*
Mn-55
Fe-56
Fe-57
Cu-63
Cu-65
Zn-66
Zn-68

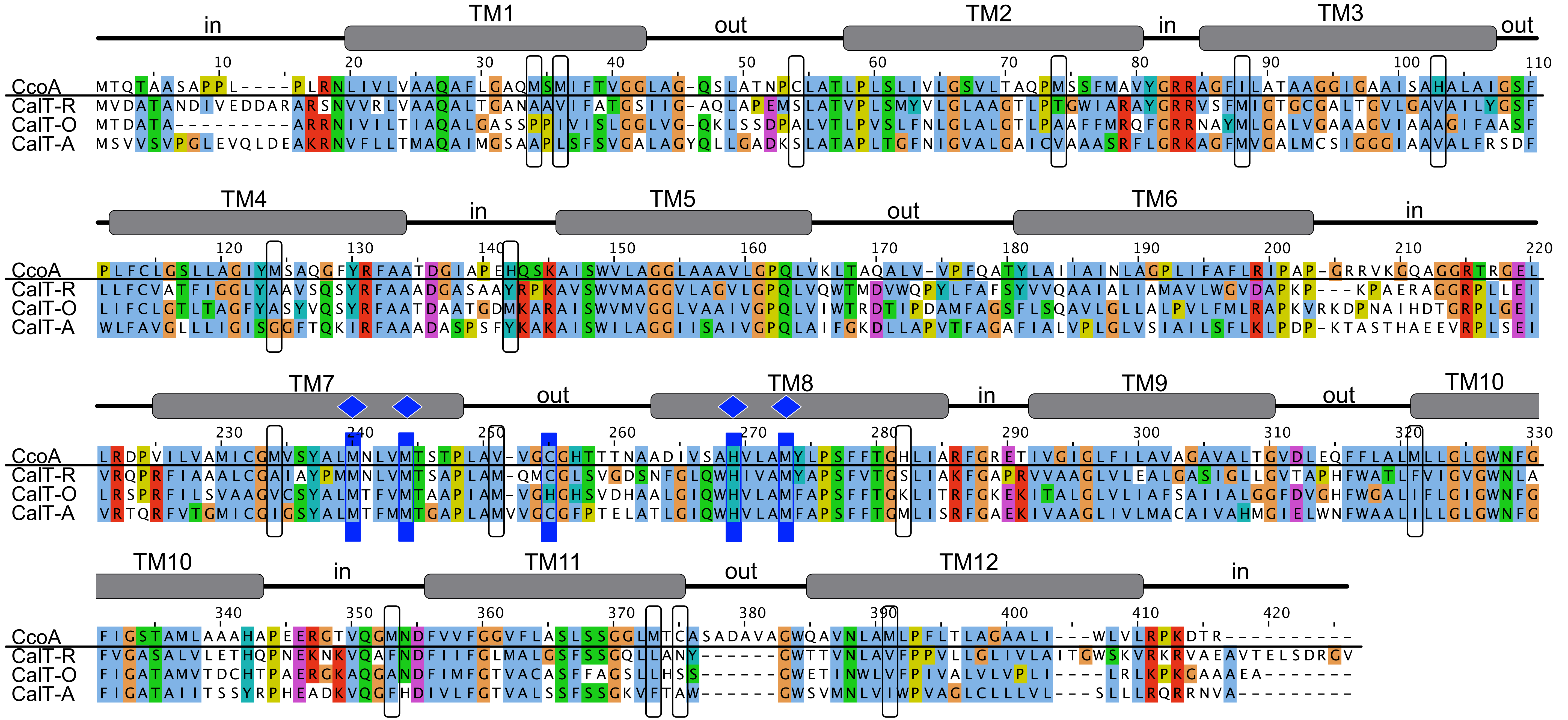

in
TM1
out
TM2
in
TM3
out
10 20 30 40 50 60 70 80 90 100 110
CcoA MTQTAASAPPL----PLRNLIVLVAAQAFLGAQMSMIFTVGGLAG-QSLATNPCLATLPLSLIVLGSVLTAQPMSSFMAVYGRRAGFILATAAGGIGAAISAHALAIGSF
CalT-R MVDATANDIVEDDARARSNVVRLVAAQALTGANAAVIFATGSIIG-AQLAPEMSLATVPLSMYVLGLAAGTLPTGWIARAYGRRVSFMIGTGCGALTGVLGAVAILYGSF
CalT-O MTDATA----------ARRNIVILTIAQALGASSPPIVISLGGLVG-QKLSSDPALVTLPVSLFNLGLALGTLPAAFFMRQFGRRNAYMLGALVGAAAGVIAAAGIFAASF
CalT-A MSVVSVPGLEVQLDEAKRNVFLLTMAQAIMGSAAPLSFSVGALAGYQLLGADKSLATAPLTGFNIGVALGAICVAAASRFLGRKAGFMVGALMCSIGGGIAAVALFRSDF
TM4
in
TM5
out
TM6
in
120 130 140 150 160 170 180 190 200 210 220
CcoA PLFCLGSLLAGIYMSAQGFYRFAATDGIAPEHQSKAISWVLAGGLAAAVLGPQLVKLTAQALV-VPFQATYLAIIAINLAGPLIFAFLRIPAP-GRRVKGQAGGRTRGEL
CalT-R LLFCVATFIGGLYAAVSQSYRFAAADGASAAYRPKAVSWVMAGGVLAGVLGPQLVQWTMDVWQPYLFAFSYVVQAAIALIAMAVLWGVDAPKP---KPAERAGGRPLLEI
CalT-O LIFCLGTLTAGFYASYVQSYRFAATDAATGDMKARAISWVMVGGLVAAIVGPQLVIWTRDTIPDAMFAGSFLSQAVLGLLALPVLFMLRAPKVRKDPNAIHDTGRPLGEI
CalT-A WLFAVGLLLIGISGGFTQKIRFAAADASPSFYKAKAISWILAGGIISAIVGPQLAIFGKDLLAPVTFAGAFIALVPLGLVSIAILSFLKLPDP-KTASTHAEEVRPLSEI
TM7
out
TM8
in
TM9
out
TM10
230 240 250 260 270 280 290 300 310 320 330
CcoA LRDPVILVAMICGMVSYALMNLVMTSTPLAV-VGCGHTTTNAADIVSAHVLAMYLPSFFTGHLIARFGRETIVGIGLFILAVAGAVALTGVDLEQFFLALMLLGLGWNFG
CalT-R VRQPRFIAAALCGAIAYPMMNLVMTSAPLAM-QMCGLSVGDSNFGLQWHIVAMYAPSFVTGSLIAKFGAPRVVAAGLVLEALGASIGLLGVTAPHFWATLFVIGVGWNLA
CalT-O LRSPRFILSVAAGVCSYALMTFVMTAAPIAM-VGHGHSVDHAALGIQWHVLAMFAPSFFTGKLITRFGKEKITALGLVLIAFSAIIALGGFDVGHFWGALIFLGIGWNFG
CalT-A VRTQRFVTGMICGIGSYALMTFMMTGAPLAMVVGCGFPTELATLGIQWHVLAMFAPSFFTGMLISRFGAEKIVAAGLIVLMACAIVAHMGIELWNFWAALILLGLGWNFG
TM10
in
TM11
out
TM12
in
340 350 360 370 380 390 400 410 420
CcoA FIGSTAMLAAAHAPEERGTVQGMNDFVVFGGVFLASLSSGGLMTCASADAVAGWQAVNLAMLPFLTLAGAALI---WLVLRPKDTR----------
CalT-R FVGASALVLETHQPNEKNKVQAFNDFIIFGLMALGSFSSGQLLANY------GWTTVNLAVFPPVLLGLIVLAITGWSKVRKRVAEAVTELSDRGV
CalT-O FIGATAMVTDCHTPAERGKAQGANDFIMFGTVACASFFAGSLLHSS------GWETINWLVFPIVALVLVPLI----LRLKPKGAAAEA-------
CalT-A FIGATAIITSSYRPHEADKVQGFHDIVLFGTVALSSFSSGKVFTAW------GWSVMNLVIWPVAGLCLLLVL---SLLLRQRRNVA---------

Marker pBAD pBK68 pYZ09 pYZ02 pYZ03 pYZ10

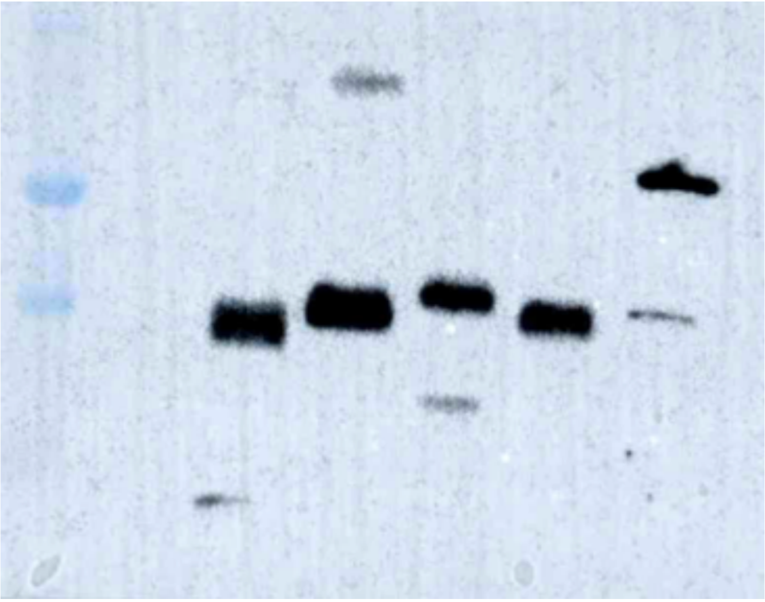

Note: lane pYZ10 is unrelated to this work on CalT, not shown in Fig 4A

Markers; 1, pBK69; 2, pYZ11; 3, pYZ07; 4,pYZ13

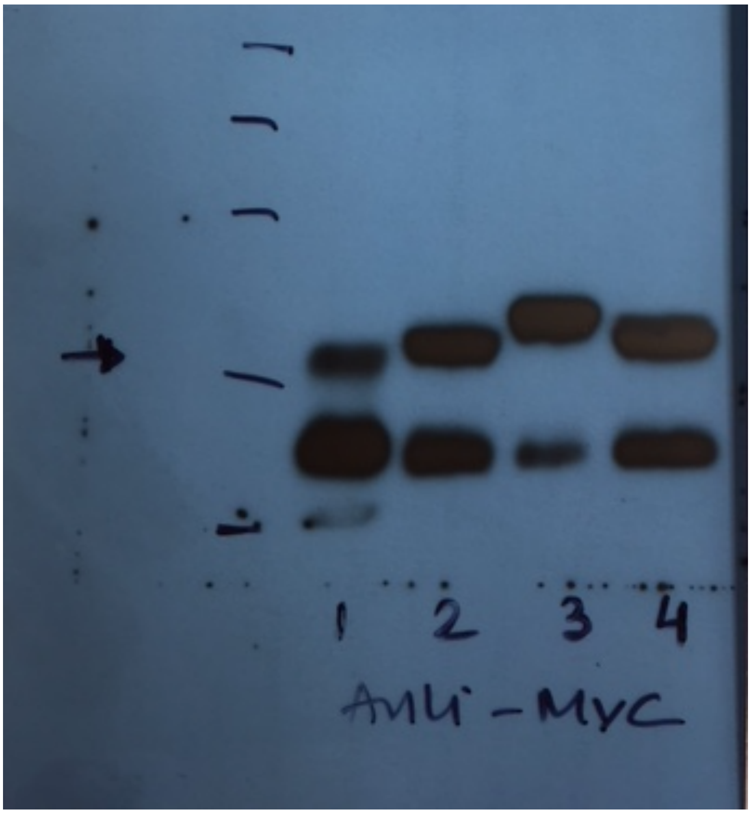

Note: lower band corresponds to peroxidase activity of c-type cyts detected by secondary antibody, unrelated to CalT, not shown in Fig 4A.

SI Table S2. Strains and plasmids used in this study

Plasmids or strains	Characteristics	Reference
Strains		
E. coli		
HB101	F-Δ(*gpt-proA*)62 *leuB6 supE44 ara*-14 *galK2 lacY*1 Δ (*mcrC-mrr*) *rpsL20* (Str^{R}) *xyl-5 mtl-1recA*13, Str^{R}	Promega
LMG194	F-Δl*acX74 galE thi rpsL* Δ*phoA* (Pvu II) Δ*ara714 leu::Tn10*, Tet^{R}	
BW25141::□*ribB*	Δ*ribB::cat*, Cm^{R} derivative of BW25141	[19,25]
R. capsulatus		
SE8	Δ*ccoA*, Spe^{R}	[13]
Plasmids		
pGRibN	pGEM-T derivative expressing *R. leguminasorum* riboflavin transporter RibN, Amp^{R}	[25]
pRK2013	Conjugation helper, Kan^{R}	[57]
pRK415	Broad-host-range plasmid, Tet^{R}	
pBAD/Myc-His A	L-Ara inducible expression plasmid, Amp^{R}	Invitrogen
pBK68	1.2 kb of *R. capsulatus ccoA* in pBAD/Myc-His A, Amp^{R}	[16]
pBK69	pBK68 cloned into pRK415 to yield a pBAD-pRK415 derivative carrying *R. capsulatus ccoA*, Amp^{R} Tet^{R}	[16]
pYZ02	1.2 kb of *R. palustris calT* (*calT-R*) in pBAD/Myc-His A, Amp^{R}	This study
pYZ03	1.2 kb of *A. tumefaciens calT* (*calT-A*) in pBAD/Myc-His	This study

	A, Amp^R	
pYZ08	Derivative of pYZ03 where the *Nsi*I site of *calT-A* was mutated, Amp^R	This study
pYZ09	1.2 kb of *O. anthropi calT (calT*-O, previously called *rfnT*) in pBAD/Myc-His A, Amp^R	This study
pYZ07	pYZ02 cloned into pRK415 to yield a pBAD-pRK415 derivative carrying *calT-R* , Amp^R Tet^R	This study
pYZ11	pYZ09 cloned into pRK415 to yield a pBAD-pRK415 derivative carrying *calT-O* (previously called *rfnT*), Amp^R Tet^R	This study
pYZ13	pYZ08 cloned into pRK415 to yield a pBAD-pRK415 derivative carrying *calT*-A, Amp^R Tet^R	This study

SI Table S3: Oligonucleotide primers used in this study

Primers	Sequence from 5’ to 3’
RPA-F	CCCAAGCTTGACGCCGCGATCGGATAGTT
RPA-R	GGGGTACCGTCGATGCGACGGCCAACGA
Atu-F	GGGGTACCAGCGTGGTTTCCGTCCCAGGAC
Atu-R	CCCAAGCTTCGCGACGTTCCTGCGCTGCCTC
OanT-F	GGGGTACCATGACCGACGCGACGGCTGC
OanT-R	CGGAATTCGCTTCGGCTGCTGCCCCTTT
AtuN-F	CGGCCGATGCCTCGCCGTCCT
AtuN-R	CCGCAAAGCGGATTTTCTGCG

SI Table S4. List of abbreviations used in the figures.

Figure 2. Phylogenetic distribution of CalT in Proteobacteria

ypfH: putative phospholipase (PF02230)

alkA: putative 3-methyladenine DNA glycosylase (PF00730)

phoX: putative glycerophosphodiester phosphodiesterase (PF13449)

UF: unknown function

btuB: similar to outer membrane B12 receptor

MFS: a member of MFS that is distinct from the CalT family members

ribD: riboflavin biosynthesis protein RibD

ribE riboflavin synthase

rhtB putative transporter

ribH riboflavin biosynthesis protein RibH

nusB global transcriptional regulator

Figure 4. Genomic neighborhoods containing CalT near putative Cu-related proteins

sdh: serine dehydratase

astE2: uncharacterized subgroup of peptidase M14 succinylglutamate desuccinylase (ASTE)/aspartoacylase (ASPA)-like

lysR: DNA-binding transcriptional regulator, *LysR* family

fer2: ferredoxin, 2Fe-2S type

sphA: (COG4313/ pfam13557), uncharacterized conserved protein

glxA: transcriptional regulator GlxA family, contains an amidase domain and an AraC-type DNA-binding HTH domain; *pncA*, nicotinamidase-related amidase

ssuD: flavin-dependent oxidoreductase, luciferase family

305: domain of unknown function (DUF305)

copA: Cu-ATPase

GRX/mauE: fusion between glutaredoxin and methylamine utilization protein MauE

csoR: metal-sensitive transcriptional repressor

cueR: MerR family regulatory protein

acrA: multidrug efflux pump subunit AcrA (membrane-fusion protein)

acrB: multidrug efflux pump subunit AcrB

tolC: outer membrane protein TolC

arsR: bacterial regulatory protein, ArsR family

442: putative phosphatase (DUF442)

cydD: (PF00005/PF00064), ABC-type transport system involved in cytochrome *bd* biosynthesis, ATPase and permease components

cydC: (PF00005), ABC-type transport system involved in cytochrome *bd* biosynthesis, fused ATPase and permease components

UF: unknown function

rimI: ribosomal protein S18 acetylase RimI and related acetyltransferases

acrR: DNA-binding transcriptional regulator, AcrR family

fer2: 2Fe-2S iron-sulfur cluster binding domain

3347: protein of unknown function (DUF3347);

rteC: (PF09357), tetracycline resistance (Tcr) element gene

3911: protein of unknown function (DUF3911)

ytkA: YtkA-like proteins from FixH family

rhod: (pfam00581), Rhodanese Homology Domain (RHOD): an alpha beta fold domain found duplicated in the rhodanese protein

crp: cAMP-binding domain of CRP or a regulatory subunit of cAMP-dependent protein kinases

cusA: Cu/Ag efflux pump CusA

cusB: CusB is a membrane fusion proteins of the CusCFBA copper efflux system in *E.coli* and related bacteria

MFS3: MFS_3 (PF05977)

hindVP: HindVP restriction endonuclease

dcm: C-5 cytosine-specific DNA methylase

omp: outer membrane protein beta-barrel domain

oep: outer membrane efflux protein;

MFS1: MFS_1 (PF07690)

oafA: peptidoglycan/LPS O-acetylase OafA/YrhL, contains acyltransferase and SGNH-hydrolase domains

bet: choline-glycine betaine transporter

cbb$_3$-Cox structural (*ccoNOQP*) and assembly (*ccoGHIS*) genes

(for *Methylosinus* sp. R-45379, *ccoQ* is likely present but not predicted during structural annotation of the corresponding genome, and for *Microvirga vignae*, *ccoS* is likely present but not predicted during structural annotation of the corresponding genome)

dsrE: intracellular sulfur oxidation protein, DsrE/DsrF family; *soxB*, sulfate thiol esterase

soxK: SoxAX-interacting protein

soxA: SoxA subunit of the SoxAX sulfite oxidase cytochrome

soxZ: sulfur compound chelating protein SoxZ

soxY: thiosulfate-binding protein SoxY

soxX: SoxX subunit of the SoxAX sulfite oxidase cytochrome

938: domain of unknown function (DUF938)

araC: contains cupin and AraC transcriptional regulator domains

soxC: sulfite dehydrogenase

gloB/pspE: fusion between GloB (a putative glyoxylase) and PspE (a putative rhodonese)

cbb$_3$: cytochrome *c*, *cbb*$_3$-type

4396: domain of unknown function (DUF4396)

putA: oxidizes proline to glutamate for use as a carbon and nitrogen source

cusF: copper binding periplasmic protein CusF

cirA: TonB dependent/Ligand-Gated channel

COG0523: family of putative GTPases involved in metal homeostasis

prmB: putative 50S ribosomal protein L3 glutamine methyltransferase

guaA: putative amidotransferase

PCuAC: copper chaperone PCu(A)C

sco1: Sco1/SenC family protein

adh: short-chain dehydrogenase family protein

cobA: tetrapyrrole (corrin/porphyrin) methylase

cutA: CutA1 divalent ion tolerance protein

Cu-SOD: superoxide dismutase, Cu-Zn family

SI Figure S2. Phylogenetic tree of RfnT-like CalT subfamily. See also the abbreviations above.

3837: COG3837 cupin superfamily

rimL: putative RimL-like acetyltransferase

coxL: putative CO or xanthine dehydrogenase, Mo-binding subunit

ycjU: member of Beta-phosphoglucomutase or related phosphatase, HAD superfamily

dgc: putative diguanylate cyclase

lysE: putative transporter

2161: DUF2161

rafL: putative O-antigen ligase

3335: putative transposase with homeodomain-like domains
